# Glc7/PP1 dephosphorylates histone H3T11 to regulate autophagy and telomere silencing in response to nutrient availability

**DOI:** 10.1038/s41421-023-00551-1

**Published:** 2023-07-11

**Authors:** Xinyu Zhang, Qi Yu, Yinsheng Wu, Yuan Zhang, Yi He, Rongsha Wang, Xilan Yu, Shanshan Li

**Affiliations:** State Key Laboratory of Biocatalysis and Enzyme Engineering, National & Local Joint Engineering Research Center of High-throughput Drug Screening Technology, School of Life Sciences, Hubei University, Wuhan, Hubei China

**Keywords:** Post-translational modifications, Histone post-translational modifications, Epigenetics

## Abstract

How cells adapt their gene expression to nutritional changes remains poorly understood. Histone H3T11 is phosphorylated by pyruvate kinase to repress gene transcription. Here, we identify the protein phosphatase 1 (PP1), Glc7 as the enzyme that specifically dephosphorylates H3T11. We also characterize two novel Glc7-containing complexes and reveal their roles in regulating gene expression upon glucose starvation. Specifically, the Glc7–Sen1 complex dephosphorylates H3T11 to activate the transcription of autophagy-related genes. The Glc7–Rif1–Rap1 complex dephosphorylates H3T11 to derepress the transcription of telomere-proximal genes. Upon glucose starvation, Glc7 expression is up-regulated and more Glc7 translocates into the nucleus to dephosphorylate H3T11, leading to induction of autophagy and derepressed transcription of telomere-proximal genes. Furthermore, the functions of PP1/Glc7 and the two Glc7-containing complexes are conserved in mammals to regulate autophagy and telomere structure. Collectively, our results reveal a novel mechanism that regulate gene expression and chromatin structure in response to glucose availability.

## Introduction

Diet and nutrients have a profound impact on human development and health^1^. Chronic exposure to nutritional deficiencies or excess triggers metabolic stress, which is closely related to the incidence and progression of many chronic diseases^1^. Metabolic stress affects gene expression pattern, resulting in cellular and physiological changes^2,3^. To cope with environmental nutrient changes and maintain cell survival, cells need to reprogram their transcriptomes but the underlying mechanisms are not well understood^4,5^.

Emerging evidence indicates that chromatin modifications are determined by cell metabolic status, which then affects gene expression and cell fates^6–8^. Cell metabolism can impact on chromatin modifications via the activity of metabolic enzymes and metabolites^9^. Several metabolites serve as substrates for chromatin modifications, such as acetyl-CoA for acetylation, S-adenosylmethionine (SAM) for methylation, and ATP for phosphorylation^10,11^. Other metabolites act as activators or inhibitors for chromatin modifying enzymes, such as α-ketoglutarate and NAD^+^^11^. Some metabolic enzymes even directly modify histones, which provides a new mechanism for specific regulation of gene expression by cell metabolism. The glycolytic enzyme, pyruvate kinase PKM2 has been reported to catalyze the phosphorylation of histone H3T11 (H3pT11) using phosphoenolpyruvate as its phosphate donor to promote gene transcription and tumorigenesis^12^. In budding yeast, pyruvate kinase Pyk1 translocates into the nucleus, where it phosphorylates H3T11 to repress gene expression within the SESAME complex (Serine-responsive SAM-containing Metabolic Enzyme)^13^. In addition, glycolysis and serine metabolism stimulate SESAME complex to phosphorylate H3T11^13,14^.

H3T11 phosphorylation has been reported to regulate the transcription of genes involved in autophagy^15,16^. Autophagy is a conserved catabolic process that is initiated with the biogenesis of autophagosomes, which then fuse with the lysosomes or vacuoles to degrade their contents. Autophagy targets cytoplasmic contents, abnormal protein aggregates and damaged organelles for degradation and recycling to maintain cellular homeostasis and promote cell survival^17^. Under nutrient starvation conditions, autophagy is induced to a high level and cells need to activate the transcription of autophagy genes to replenish autophagy components^18,19^. The SESAME complex phosphorylates H3T11 at autophagy-related genes to repress their transcription and inhibit autophagy^15,16^. As SESAME-catalyzed H3T11 phosphorylation is enhanced with the increasing glucose concentrations, the autophagy activity is maintained at basal low levels under nutrient-rich conditions^15^.

H3T11 phosphorylation has also been reported to maintain gene silencing near telomere regions^15^. Telomere has a typical heterochromatin structure and genes near these regions are subject to transcriptional repression, also known as telomere silencing^20,21^. Maintaining the integrity of telomere silencing is required for cell growth, development and differentiation^22–24^. Telomere heterochromatin is primarily maintained by assembly of the SIR (Silent Information Regulator) complex (Sir2/Sir3/Sir4), which binds at telomere regions and makes the underlying DNA inaccessible from the transcription machinery^20,21^. H3T11 phosphorylation enhances the SIR complex binding at telomere regions^15^. Moreover, H3T11 phosphorylation prevents autophagy-mediated degradation of Sir2, which further facilitates the binding of SIR complex at telomeres^15^. Although H3T11 phosphorylation directly connects autophagy with telomere silencing, how H3T11 phosphorylation is removed and dynamically regulated in response to nutritional changes remains largely unknown.

Glc7 is the sole catalytic subunit of protein phosphatase 1 (PP1), which was initially identified to regulate glycogen metabolism in budding yeast^25^. Glc7 has been reported to play important roles in regulating vesicle trafficking, cell polarity, DNA damage, transcription termination, cell-cycle progression, and etc.^26–28^. In this study, we identified histone H3T11 as a novel target for Glc7/PP1 and characterized two conserved Glc7-containing complexes that coordinately regulate autophagy and telomere silencing. Moreover, the activity of Glc7/PP1 to dephosphorylate H3T11 was enhanced under glucose starvation, resulting in induction of autophagy and reduction of telomere silencing. Most importantly, the above two Glc7-containing complexes are conserved in mammalian cells to regulate autophagy, telomere silencing and cellular senescence. Collectively, our study reveals that H3T11 phosphorylation is dynamically regulated by the highly conserved PP1 enzyme system, providing a mechanism by which cells regulate gene expression and chromatin structure in response to glucose availability.

## Results

### Glc7/PP1 dephosphorylates histone H3T11

**Fig. 1:**
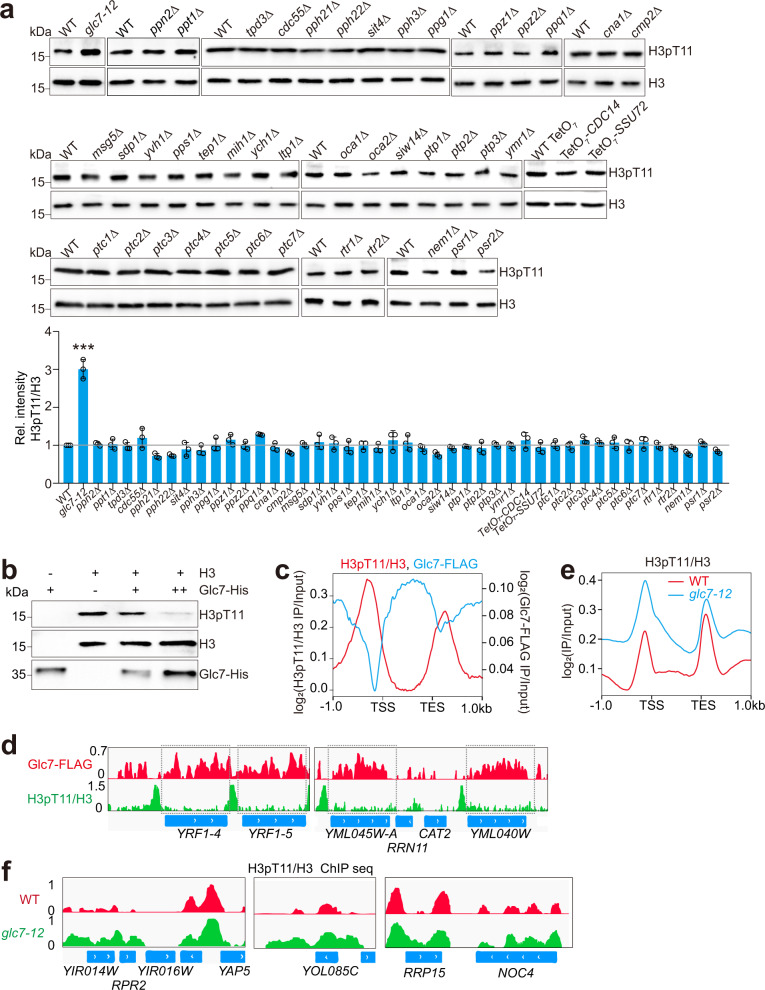
Glc7 dephosphorylates H3T11 both in vivo and in vitro. **a** Immunoblot analysis of the intracellular H3pT11 levels in 44 protein phosphatase mutants. The relative intensities of H3pT11/H3 were quantified using Image J with standard error (SE). Yeast cells were grown in YPD medium at 30 °C until OD_600_ of 1.0. To inactivate Glc7, WT and *glc7-12* mutant were grown at 26 °C until OD_600_ of 0.5 and then treated at 37 °C for 2 h. For WT TetO_7_, TetO_7_-*CDC14*, and TetO_7_-*SSU72*, cells were grown in YPD medium at 30 °C until OD_600_ of 0.5–0.7 and then treated with 40 μg/mL doxycycline for 2 h to knockdown the expression of *CDC14* and *SSU72*, respectively. **b** In vitro dephosphorylation assay showing purified recombinant Glc7-His dephosphorylates histone H3T11. **c** Distribution of H3pT11/H3 and Glc7-FLAG across each gene through 1 kb upstream of the TSS to 1 kb downstream from the TES. The ChIP-seq were performed when cells were grown at 30 °C. Glc7-FLAG ChIP was performed with anti-FLAG antibody. Log_2_ ratios of H3pT11/H3 and Glc7-FLAG IP/Input at significantly enriched windows were used. **d** ChIP-seq tracks showing the occupancy of Glc7-FLAG and H3pT11/H3 at representative genes. **e** Distribution of H3pT11/H3 across each gene through 1 kb upstream of the TSS to 1 kb downstream from the TES in WT and *glc7-12* mutant. WT and *glc7-12* mutant were grown at 37 °C to inactivate Glc7. Log_2_ ratios of H3pT11/H3 at significantly enriched windows were used. **f** ChIP-seq tracks showing the enrichment of H3pT11/H3 at representative genes in WT and *glc7-12* mutant. For **a**, data represent the mean ± SE of three biological independent experiments. *P < 0.05, **P < 0.01, ***P < 0.001.

To identify the phosphatase(s) that removes H3T11 phosphorylation (H3pT11), we first set out to screen the yeast protein phosphatase mutant library with anti-H3pT11 antibody. This library contains a total of 44 protein phosphatase mutants, including PPP family (Glc7, Ppn2, Ppt1, Tpd3, Cdc55, Pph21, Pph22, Sit4, Pph3, Ppg1, Ppz1, Ppz2, Ppq1, Cna1, Cmp2), PTP family (Msg5, Sdp1, Yvh1, Pps1, Tep1, Mih1, Ych1, Ltp1, Oca1, Oca2, Siw14, Ptp1, Ptp2, Ptp3, Ymr1, Cdc14, Ssu72), PPM family (Ptc1, Ptc2, Ptc3, Ptc4, Ptc5, Ptc6, Ptc7), RTR family (Rtr1, Rtr2), and HAD family (Nem1, Psr1, Psr2)^29,30^. Among these protein phosphatase mutants, inactivation of PP1 phosphatase Glc7 in a temperature-sensitive mutant (*glc7-12*) at a non-permissive temperature (37 °C)^31^ significantly increased the intracellular H3pT11 (Fig. 1a; Supplementary Fig. S1a). No significant changes of H3pT11 were observed in the mutants of other protein phosphatases (Fig. 1a). The molecular docking assay revealed that the phosphorylated H3T11 was in close proximity to Glc7 catalytic domain (Supplementary Fig. S1b). To directly show that Glc7 can dephosphorylate H3T11, we purified Glc7 by tandem affinity purification (Glc7-CBP) from yeast cells and performed in vitro dephosphorylation assay with in vitro-assembled nucleosomes that were pre-phosphorylated at H3T11. This assay revealed that Glc7 directly reduced H3pT11 at nucleosomes (Supplementary Fig. S1c). Similar dephosphorylation activity was observed with purified recombinant Glc7 (Glc7-His) and recombinant histone H3 (Fig. 1b; Supplementary Fig. S1d). As *GLC7* encodes the sole PP1 catalytic subunit in budding yeast^26^, these data indicate that Glc7/PP1 is the primary protein phosphatase for H3T11.

We then performed chromatin immunoprecipitation coupled with high throughput sequencing (ChIP-seq) for Glc7. By comparing the ChIP-seq data for Glc7 and H3pT11^15^, we observed that Glc7 preferentially bound to gene coding regions, whereas H3pT11 was primarily enriched at upstream of the transcription start site (TSS) and downstream from the transcription end site (TES) (Fig. 1c, d; Supplementary Fig. S1e). Hence, the binding pattern of Glc7 anti-correlates with the enrichment of H3pT11 genome-wide. We also performed ChIP-seq for H3pT11 in wild-type (WT) and *glc7-12* mutant when cells were grown at 37 °C to inactivate Glc7. The occupancy of H3pT11 at chromatin was markedly increased in *glc7-12* mutant when compared with WT (Fig. 1e, f), further supporting the concept that Glc7 dephosphorylates H3T11 in vivo. We also noted that H3pT11 was increased between genes in *glc7-12* mutant (Fig. 1e, f), which may be caused by the effect of Glc7 on gene transcription. Collectively, we identified Glc7/PP1 as the primary phosphatase that mediates the dephosphorylation of histone H3T11.

### Glc7/PP1 dephosphorylates H3T11 to transcriptionally activate autophagy

**Fig. 2:**
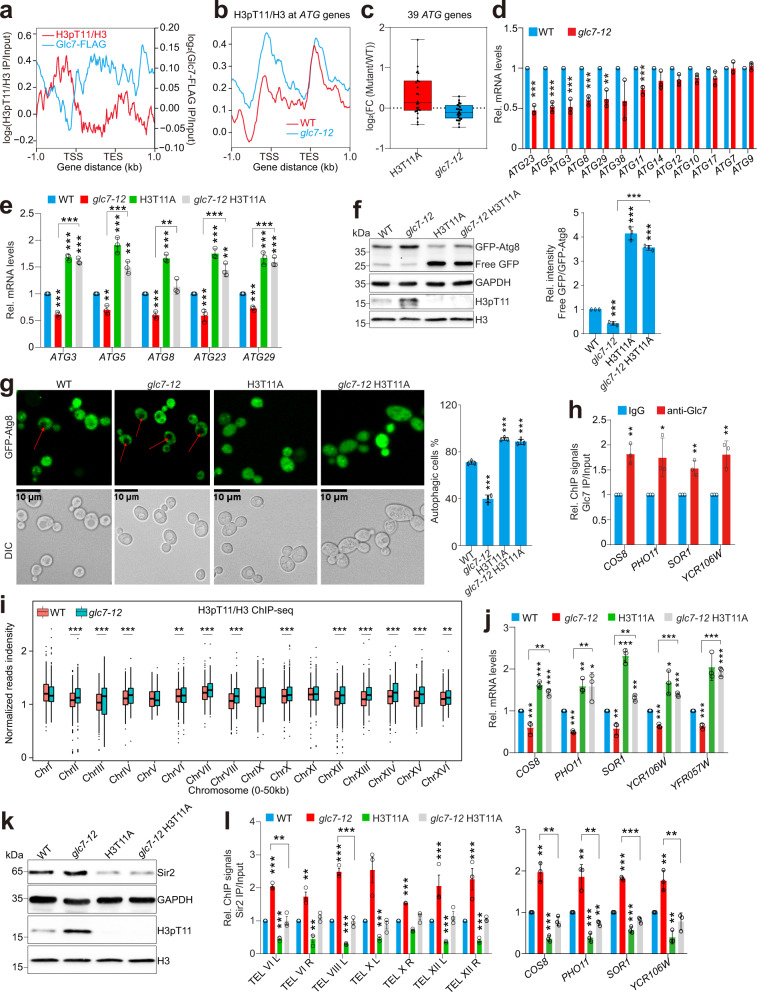
Glc7 dephosphorylates H3T11 to regulate autophagy and telomere silencing. **a** Distribution of H3pT11/H3 and Glc7-FLAG across 39 *ATG* genes through 1 kb upstream of the TSS to 1 kb downstream from the TES. Log_2_ ratios of H3pT11/H3 and Glc7-FLAG IP/Input at significantly enriched windows were used. **b** Distribution of H3pT11/H3 across 39 *ATG* genes through 1 kb upstream of the TSS to 1 kb downstream from the TES in WT and *glc7-12* mutant. **c** Box plot showing the transcriptional changes of 39 *ATG* genes in H3T11A and *glc7-12* mutants. FC, fold change. **d** RT-qPCR analysis of *ATG* gene transcription in WT and *glc7-12* mutant. **e** RT-qPCR analysis of *ATG* gene transcription in WT, *glc7-12*, H3T11A and *glc7-12* H3T11A mutants. **f**, **g** Analysis of the autophagy activity in WT, *glc7-12*, H3T11A and *glc7-12* H3T11A mutants as determined by GFP-Atg8 processing assay (**f**) and fluorescence assay (**g**). The vacuoles of yeast cells were indicated with red arrows. **h** ChIP-qPCR analysis of Glc7 occupancy (anti-Glc7 antibody) at telomere-proximal genes. IgG was used as a control. **i** Box plots showing the effect of Glc7 inactivation in *glc7-12* mutant on H3pT11 occupancy at subtelomeric regions on 16 chromosomes. The subtelomeric regions refer to 0–50 kb from the nearest left or right telomeres. **j** RT-qPCR analysis of the transcription of telomere-proximal genes in WT, *glc7-12*, H3T11A and *glc7-12* H3T11A mutants. **k** Immunoblots of Sir2 in WT, *glc7-12*, H3T11A and *glc7-12* H3T11A mutants. **l** ChIP-qPCR analysis of the binding of Sir2 (anti-Sir2 antibody) at subtelomere regions and telomere-proximal genes in WT, *glc7-12*, H3T11A and *glc7-12* H3T11A mutants. For **d**–**g**, **i**–**l**, cells were grown in YPD medium at 26 °C until OD_600_ of 0.5 and then treated at 37 °C for 2 h to inactivate Glc7. For **d**–**h**, **j**, **l**, data represent the mean ± SE of three biological independent experiments. *P < 0.05, **P < 0.01, ***P < 0.001.

H3T11 phosphorylation has been reported to transcriptionally repress autophagy^15^. We first analyzed the ChIP-seq for the occupancy of Glc7 and H3pT11 at 39 autophagy-related genes. There was an anti-correlation between the occupancy of Glc7 and H3pT11 at *ATG* genes (Fig. 2a; Supplementary Fig. S2a). The occupancy of Glc7 at *ATG* genes (*ATG5*, *ATG8*, *ATG23*) was confirmed by ChIP-qPCR (Supplementary Fig. S2b). The occupancy of H3pT11 at *ATG* genes was significantly increased in *glc7-12* mutant (Fig. 2b; Supplementary Fig. S2c). We then examined the autophagy activity in *glc7-12* mutant using the GFP-Atg8 processing assay, which is based on the principle that upon autophagy induction, GFP-Atg8 is transported into the vacuoles, where the Atg8 portion is degraded with the free GFP moiety resistant to proteolysis^32^. The ratio of free GFP/GFP-Atg8 hence highly correlates with the autophagic flux^32^. Inactivation of Glc7 significantly reduced autophagy flux as indicated by decreased ratio of free GFP/GFP-Atg8 (Supplementary Fig. S2d).

Next, we performed RNA sequencing (RNA-seq) for WT, H3T11A and *glc7-12* mutants to examine the effect of Glc7 on transcription of autophagy-related genes. Most *ATG* genes were down-regulated in *glc7-12* mutant, which is in contrast to H3T11A mutant (Fig. 2c; Supplementary Fig. S2e). The down-regulation of *ATG* genes in *glc7-12* mutant was confirmed by RT-qPCR (Fig. 2d), indicating that Glc7 activates the transcription of autophagy-related genes.

To determine whether Glc7 activates the transcription of *ATG* genes by dephosphorylating H3T11, we mutated H3T11A in *glc7-12* mutant to construct *glc7-12* H3T11A double mutant. The transcription of *ATG* genes was significantly reduced in *glc7-12* mutant but was significantly increased in *glc7-12* H3T11A mutant (Fig. 2e). In line with the changes of *ATG* gene expression, the autophagy activity was significantly reduced in *glc7-12* mutant but mutation of H3T11A significantly increased the autophagy activity in *glc7-12* mutant (Fig. 2f). To strengthen these findings, we examined the autophagy-dependent translocation of GFP-Atg8 to the vacuole by fluorescence microscopy. The percentage of autophagic cells that displayed clearly vacuolar localization of GFP was significantly decreased in *glc7-12* mutant but was significantly increased in *glc7-12* H3T11A mutant (Fig. 2g). These data suggest that Glc7/PP1 dephosphorylates H3T11 to activate autophagy.

Glc7 has been reported to dephosphorylate histone H3S10 during mitosis^33^. We examined the effect of H3S10A mutation on *ATG* gene transcription. Ablation of H3S10 phosphorylation in H3S10A mutant had no significant effect on transcription of *ATG* genes that were activated by Glc7 (Supplementary Fig. S2f). Moreover, mutation of H3S10A did not significantly affect the autophagy activity (Supplementary Fig. S2g). These data indicate that Glc7/PP1 activates autophagy independent of H3S10 phosphorylation.

### Glc7/PP1 dephosphorylates H3T11 to reduce telomere silencing

H3T11 phosphorylation has been reported to connect autophagy with telomere silencing. H3T11 phosphorylation inhibits the autophagy-mediated degradation of Sir2 to promote the binding of Sir2/Sir3/Sir4 complex at telomere regions^15^. Meanwhile, H3T11 phosphorylation can directly enhance the binding of Sir2/Sir3/Sir4 complex at telomere regions^15,16^. By analyzing the ChIP-seq data for Glc7, we observed the binding of Glc7 at subtelomere regions and its binding pattern was still opposite to that of H3pT11 (Supplementary Fig. S3a). The binding of Glc7 at subtelomere regions was further confirmed by ChIP-qPCR (Fig. 2h; Supplementary Fig. S3b). Inactivation of Glc7 significantly increased the occupancy of H3pT11 at most subtelomere regions, which was confirmed by ChIP-qPCR (Fig. 2i; Supplementary Fig. S3c). For some subtelomere regions, such as ChrI, ChrV, ChrIX and ChrXI, H3pT11 at these regions was not significantly increased in *glc7-12* mutant (Fig. 2i), which may be caused by lower binding of Glc7. It could also be caused by the action of other protein phosphatase(s) in *glc7-12* mutant. Inactivation of Glc7 also significantly increased the occupancy of H3pT11 at telomere-proximal genes, including *YFR057W*, *YCR106W*, *PHO11*, *COS8*, *SEO1*, and *SOR1*, which locate 0.64 kb, 3.2 kb, 3.4 kb, 6.4 kb, 7.2 kb, and 8.6 kb away from the nearest telomeres, respectively (Supplementary Fig. S3d)^15^. Accordingly, inactivation of Glc7 significantly reduced the transcription of these telomere-proximal genes (Supplementary Fig. S3e). Moreover, analysis of the RNA-seq data for *glc7-12* mutant revealed that there was a striking subtelomere bias for genes repressed in *glc7-12* mutant: the fraction of down-regulated genes within 30 kb of the chromosome ends was significantly higher than the genome-wide average as determined by a *χ*^*2*^ test (Supplementary Fig. S3f). These data suggest a direct role of Glc7/PP1 in derepressing the transcription of telomere-proximal genes.

To address whether Glc7 reduces telomere silencing via dephosphorylation of H3T11, we examined the transcription of telomere-proximal genes in WT, H3T11A, *glc7-12* and *glc7-12* H3T11A mutants by RT-qPCR. The transcription of telomere-proximal genes was significantly decreased in *glc7-12* mutant but was increased in *glc7-12* H3T11A mutant (Fig. 2j). In contrast, the transcription of these telomere-proximal genes was unaffected in H3S10A mutant (Supplementary Fig. S3g), suggesting that Glc7/PP1 reduces telomere silencing independently of H3S10 dephosphorylation.

H3T11 phosphorylation has been reported to prevent the nucleus export of Sir2 and protect Sir2 from being degraded by autophagy^15^. We thus examined the effect of Glc7 on Sir2 protein levels. Inactivation of Glc7 significantly increased the global Sir2 protein levels (Supplementary Fig. S3h). When WT and *glc7-12* mutant were treated with rapamycin, an mTOR kinase inhibitor to induce autophagy, the overall Sir2 protein levels were higher in *glc7-12* than those in WT (Supplementary Fig. S3i). The occupancy of Sir2 at subtelomere regions and telomere-proximal genes was significantly increased in *glc7-12* mutant (Supplementary Fig. S3j). In addition, more Sir2 was localized in the nucleus in *glc7-12* mutant than WT (Supplementary Fig. S3k), consistent with the role of H3T11 phosphorylation in restraining the nucleus export of Sir2^15^. To determine whether Glc7 reduces Sir2 protein levels by dephosphorylating H3T11, we examined Sir2 protein levels in WT, *glc7-12*, H3T11A, and *glc7-12* H3T11A mutants. Sir2 was reduced in H3T11A mutant, but was increased in *glc7-12* mutant (Fig. 2k). Mutation of H3T11A reduced Sir2 protein levels in *glc7-12* mutant (Fig. 2k), suggesting that inactivation of Glc7 increases H3T11 phosphorylation and represses the autophagy-mediated Sir2 degradation. The occupancy of Sir2 at subtelomere regions was significantly increased in *glc7-12* mutant but mutation of H3T11A reduced the occupancy of Sir2 to normal levels in *glc7-12* mutant (Fig. 2l). Although Sir2 protein levels were similar in H3T11A and *glc7-12* H3T11A mutants (Fig. 2k), the Sir2 occupancy at *glc7-12* H3T11A mutant is slightly higher than that in H3T11A mutant (Fig. 2l), suggesting that Glc7 may regulate Sir2 binding not only by affecting its stability but also by other mechanism. For example, Glc7 may regulate the phosphorylation of Sir2 to influence its binding to chromatin.

Collectively, these data suggest two novel functions of Glc7 in regulating telomere silencing: on one hand, Glc7 dephosphorylates H3T11 at subtelomere regions, which leads to dissociation of the SIR complex at telomeres; on the other hand, Glc7 dephosphorylates H3T11 at autophagy-related genes to enhance autophagy activity and accelerate autophagy-mediated Sir2 degradation, which further reduces Sir2 binding at subtelomere regions and decreases telomere silencing.

### Sen1 recruits Glc7/PP1 to dephosphorylate H3T11 at autophagy-related genes

**Fig. 3:**
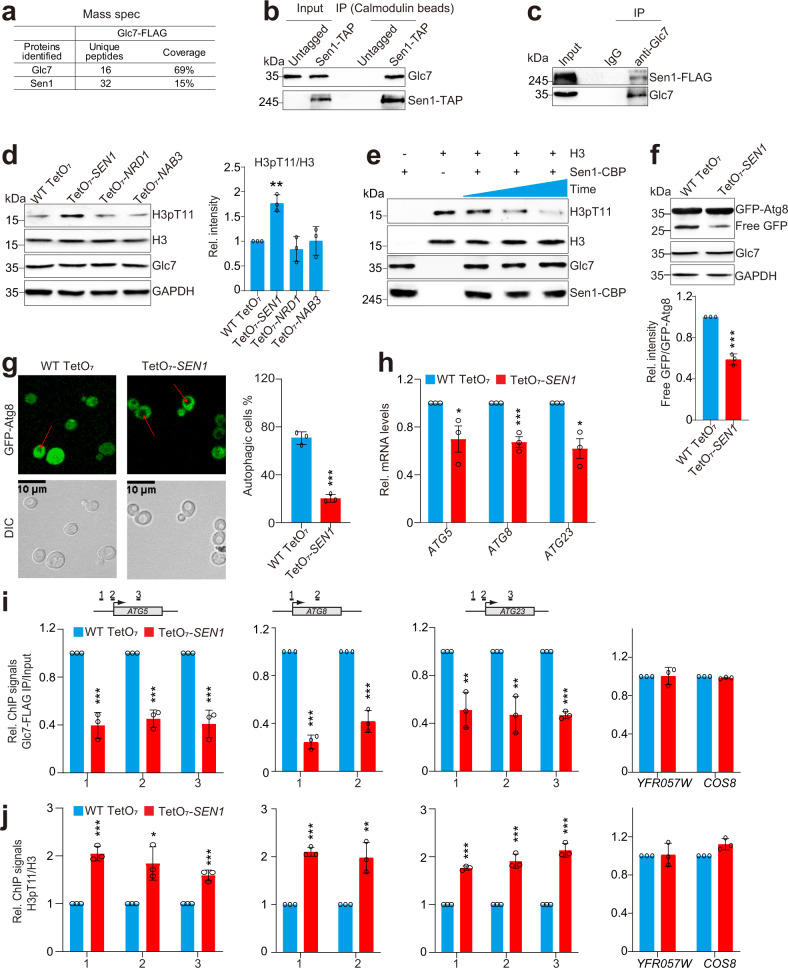
Sen1 recruits Glc7 to dephosphorylate H3T11 at autophagy-related genes. **a** IP-MS analysis of proteins co-purified with Glc7-FLAG. The endogenously expressed Glc7-FLAG was immunoprecipitated from yeast cells with anti-FLAG agarose beads. The identified unique peptides and sequence coverage were listed. **b** The endogenously expressed Sen1 interacted with Glc7 as determined by Co-IP assay. Sen1-TAP was immunoprecipitated with calmodulin beads. The co-immunoprecipitated proteins were detected with indicated antibodies. **c** Reciprocal Co-IP assay showing that endogenous Glc7 interacts with Sen1. Glc7 was immunoprecipitated with anti-Glc7 antibody. The co-immunoprecipitated proteins were detected with indicated antibodies. IgG was used as a negative control. **d** Immunoblot analysis of intracellular H3pT11 levels in WT TetO_7_, TetO_7_-*SEN1*, TetO_7_-*NRD1* and TetO_7_-*NAB3* mutants. WT TetO_7_, TetO_7_-*SEN1*, TetO_7_-*NRD1* and TetO_7_-*NAB3* mutants were grown in YPD medium at 30 °C until OD_600_ of 0.5–0.7 and then treated with 40 μg/mL doxycycline for 2 h to knockdown the expression of *SEN1*, *NRD1* and *NAB3*, respectively. The knockdown efficiency results were shown in Supplementary Fig. S4b. **e** The purified Sen1 complex (Sen1-CBP) dephosphorylates H3T11 as determined by in vitro dephosphorylation assay. **f**, **g** Analysis the effect of Sen1 knockdown on autophagy activity as determined by GFP-Atg8 processing assay (**f**) and fluorescence assay (**g**). The vacuoles of yeast cells were indicated with red arrows. **h** RT-qPCR analysis of *ATG* gene transcription in WT TetO_7_ and TetO_7_-*SEN1* mutant. **i**, **j** ChIP-qPCR analysis of the occupancy of Glc7 (**i**) and H3pT11/H3 (**j**) at *ATG* genes in WT TetO_7_ and TetO_7_-*SEN1* mutant. For **f**–**j**, WT TetO_7_ and TetO_7_-*SEN1* mutant were grown in YPD medium at 30 °C until OD_600_ of 0.5–0.7 and then treated with 40 μg/mL doxycycline for 2 h to knockdown the expression of *SEN1*. Data represent the mean ± SE of three biological independent experiments. *P < 0.05, **P < 0.01, ***P < 0.001.

Glc7/PP1 is targeted to appropriate substrates via its interaction with different regulators^27^. Reg1 is a regulatory subunit of Glc7 that targets its activity to proteins involved in the glucose repression pathway^34^. Ypi1 and Sds22 are also regulatory subunits of Glc7^35^. However, deletion of *REG1* or knockdown of *YPI1* and *SDS22* did not affect the intracellular H3pT11 (Supplementary Fig. S4a). To understand how Glc7 is recruited to chromatin to dephosphorylate H3T11, we immunoprecipitated the endogenous FLAG-tagged Glc7 (Glc7-FLAG) from yeast cells, and mass spectrometry (IP-MS) analysis detected the presence of chromatin-associated protein, Sen1 (Fig. 3a). The interaction between the endogenously expressed Glc7 and Sen1 was confirmed by co-immunoprecipitation (Co-IP) assay and reciprocal IP (Fig. 3b, c). As *SEN1* is an essential gene, we employed the promoter-shutoff strain, TetO_7_-*SEN1*, in which the *SEN1* promoter was replaced with TetO_7_, whose transcription can be shut off by adding doxycycline^36^ (Supplementary Fig. S4b). Knockdown of *SEN1* significantly increased the intracellular H3pT11 (Fig. 3d). Sen1 has been reported to be present in a complex with Nrd1 and Nab3 to regulate the transcription termination of small nucleolar RNAs^37^. Although Glc7 interacted with Nrd1 and Nab3 (Supplementary Fig. S4c–f), knockdown of Nrd1 and Nab3 had no significant effect on intracellular H3pT11 (Fig. 3d; Supplementary Fig. S4b). We then purified Sen1 complex by tandem affinity purification (Sen1-CBP) from yeast cells and performed the in vitro dephosphorylation assay. Glc7 was co-purified with Sen1 complex, which directly dephosphorylated H3T11 in a time-dependent manner (Fig. 3e). We also examined whether the helicase activity of Sen1 is important for the phosphatase activity of Sen1–Glc7 complex. We mutated Sen1 G1747D in the essential helicase domain, which has been reported to reduce its helicase activity^38^. Mutation of Sen1 G1747D had no significant effect on intracellular H3pT11 levels (Supplementary Fig. S4g). Moreover, we performed in vitro dephosphorylation assay with purified WT Sen1 and Sen1 G1747D complex. Both WT Sen1-CBP and Sen1 G1747D-CBP displayed similar phosphatase activity (Supplementary Fig. S4h), indicating that the DNA helicase activity of Sen1 is not required for the phosphatase activity of Sen1–Glc7 complex.

We then examined the effect of Sen1–Glc7 on autophagy activity. Similar to inactivation of Glc7, knockdown of *SEN1* significantly reduced autophagy activity (Fig. 3f, g). The transcription of *ATG* genes was also significantly reduced in *SEN1*-knockdown cells (Fig. 3h). In addition, knockdown of *SEN1* significantly reduced the occupancy of Glc7 at *ATG* genes (Fig. 3i), indicating that Sen1 targets Glc7 to autophagy-related genes. In accordance with the reduced Glc7 binding, the enrichment of H3pT11 at *ATG* genes was significantly increased in *SEN1*-knockdown cells (Fig. 3j). In addition to recruiting Glc7 to *ATG* genes, Sen1 enhanced the binding of Glc7 to nucleosomes as knockdown of Sen1 reduced the interaction between Glc7 and nucleosomes (Supplementary Fig. S4i). Collectively, these data indicate that Sen1 and Glc7 form a complex to dephosphorylate H3T11 at autophagy-related genes and transcriptionally activate autophagy.

### Rap1 and Rif1 recruit Glc7/PP1 to telomere regions to dephosphorylate H3T11

**Fig. 4:**
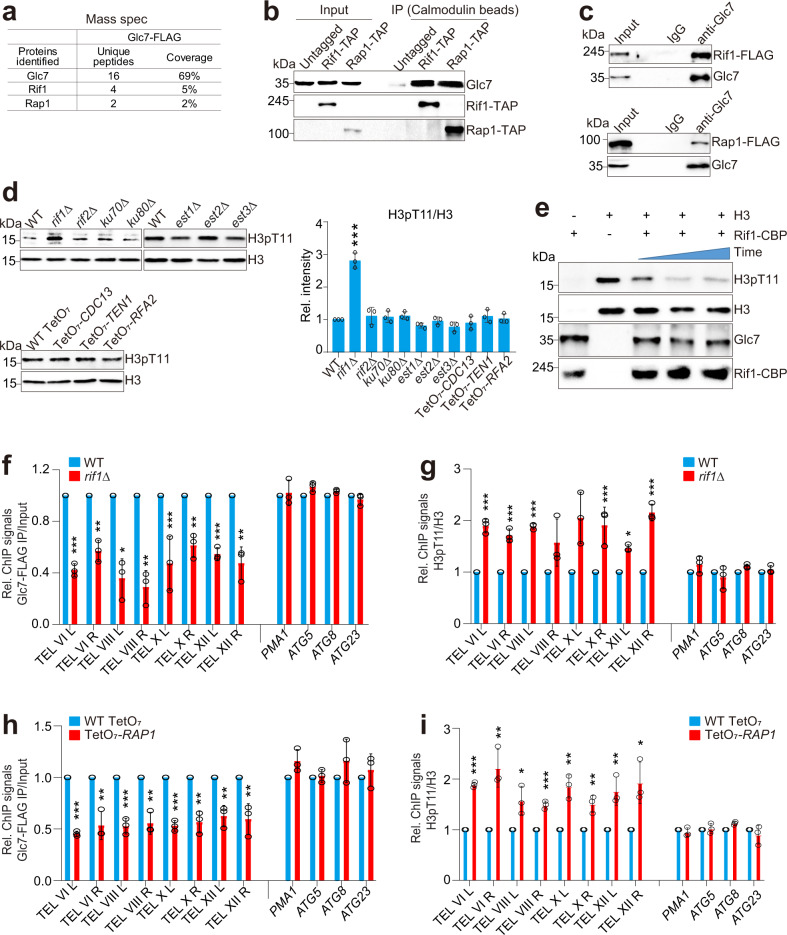
Rif1 and Rap1 recruit Glc7 to subtelomere regions to dephosphorylate H3T11. **a** IP-MS analysis of proteins co-purified with Glc7-FLAG. The identified unique peptides and sequence coverage were listed. **b**, **c** Co-IP and reciprocal IP showing that endogenously expressed Glc7 interacts with Rif1 and Rap1. **d** Immunoblot analysis of intracellular H3pT11 levels in the mutants of the indicated telomere-binding factors. WT TetO_7_, TetO_7_-*CDC13*, TetO_7_-*TEN1* and TetO_7_-*RFA2* mutants were grown in YPD medium at 30 °C until OD_600_ of 0.5–0.7 and then treated with 40 μg/mL doxycycline for 2 h to knockdown the expression of *CDC13*, *TEN1* and *RFA2*, respectively. The knockdown efficiency results were shown in Supplementary Fig. S4b. **e** The purified Rif1 complex (Rif1-CBP) dephosphorylates H3T11 as determined by in vitro dephosphorylation assay. **f**, **g** ChIP-qPCR analysis of the occupancy of Glc7-FLAG (**f**) and H3pT11/H3 (**g**) at subtelomere regions and telomere-proximal genes in WT and *rif1Δ* mutant. The autophagy-related genes and *PMA1* were used as controls. **h**, **i** ChIP-qPCR analysis of the effect of Rap1 knockdown on the occupancy of Glc7-FLAG (**h**) and H3pT11/H3 (**i**). WT TetO_7_ and TetO_7_-*RAP1* mutant treated with 40 μg/mL doxycycline for 2 h. For **d**, **f**–**i**, data represent the mean ± SE of three biological independent experiments. *P < 0.05, **P < 0.01, ***P < 0.001.

Although Sen1 recruits Glc7 to dephosphorylate H3T11 at autophagy-related genes, it had no significant effect on the occupancy of Glc7 and H3pT11 at telomere-proximal genes (Fig. 3i, j), suggesting that Glc7 is recruited to subtelomere regions by other regulators. We resorted to the IP-MS analysis to identify its interaction partners. Among the IP-MS hits are two telomere-binding proteins, Rap1 and Rap1-interacting factor 1 (Rif1) (Fig. 4a). Rap1 is a DNA-binding protein that recruits Rif1 and Rif2 to telomere regions to maintain telomere length^39–41^. The interaction between endogenous Glc7, Rif1 and Rap1 was confirmed by Co-IP and reciprocal IP (Fig. 4b, c). This interaction is specific as Glc7 had no interaction with other telomere-associated factors, such as Rif2, Reb1 and Cdc13 (Supplementary Fig. S5a), which are involved in regulation of telomere silencing and/or telomere elongation^42–45^. The intracellular H3pT11 was significantly increased in *rif1Δ* mutant (Fig. 4d). We also purified Rif1 by tandem affinity purification (Rif1-CBP) from yeast cells and performed the in vitro dephosphorylation assay. Glc7 was co-purified with Rif1 protein, which directly dephosphorylated histone H3T11 in a time-dependent manner (Fig. 4e).

We then examined the effect of Rif1 on Glc7 occupancy at subtelomere regions. Loss of Rif1 significantly reduced the occupancy of Glc7 at subtelomere regions but not at *ATG* genes (Fig. 4f). In accordance with reduced Glc7 binding, the enrichment of H3pT11 at subtelomere regions was significantly increased in *rif1Δ* mutant (Fig. 4g). Similarly, knockdown of *RAP1* significantly reduced the binding of Glc7 and increased the occupancy of H3pT11 at subtelomere regions (Fig. 4h, i). These data indicate that Rap1 and Rif1 recruit Glc7 (Glc7–Rif1–Rap1) to subtelomere regions to dephosphorylate H3T11. We also constructed TetO_7_*-SEN1 rif1Δ* double mutant. The global H3pT11 level was significantly increased in TetO_7_*-SEN1 rif1Δ* double mutant when compared with TetO_7_*-SEN1* and *rif1Δ* mutants (Supplementary Fig. S5b). Moreover, the Co-IP assay showed that Sen1 had no interaction with Rif1 or Rap1 (Supplementary Fig. S5c), further supporting that Glc7 forms two distinct complexes to dephosphorylate H3T11 at different chromatin regions.

### Glc7/PP1 dephosphorylates H3T11 to induce autophagy and reduce telomere silencing upon glucose starvation

**Fig. 5:**
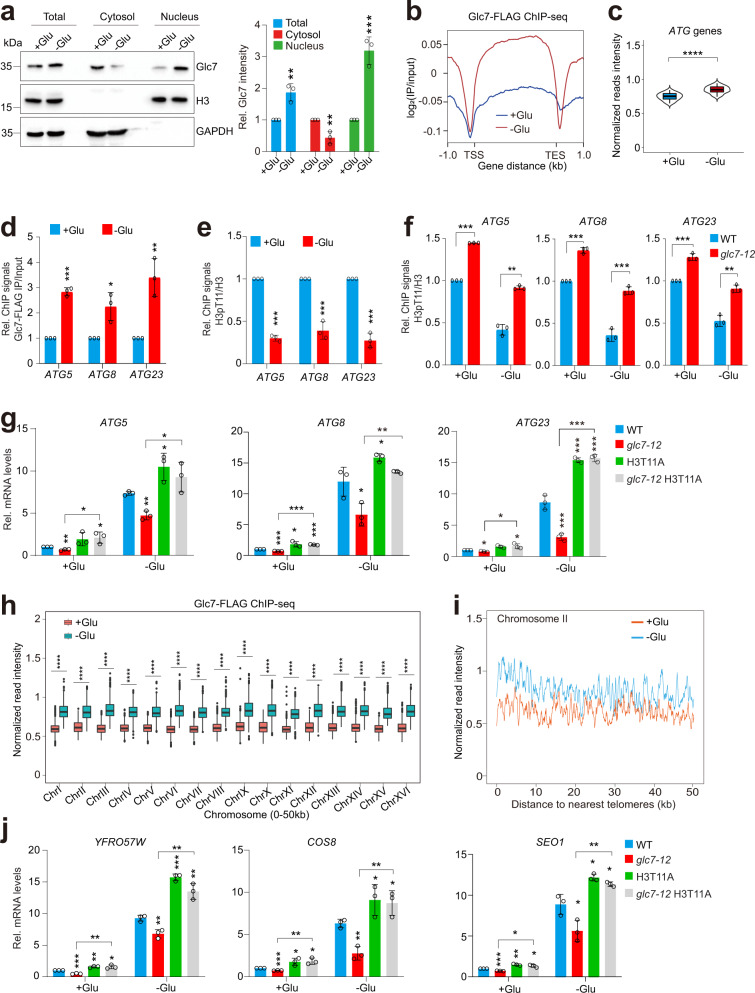
Glc7 dephosphorylates histone H3T11 to induce autophagy and reduce telomere silencing under glucose starvation conditions. **a** Subcellular fractionation assay showing the effect of glucose starvation on the localization of Glc7 in the cytoplasm and nucleus. Cells were grown in YPD medium until OD_600_ of 0.7, harvested, and then grown in SD (+Glu) medium or SD-C (−Glu) medium for 2 h. **b** ChIP-seq analysis of Glc7-FLAG occupancy across each gene through 1 kb upstream of the TSS to 1 kb downstream from the TES when cells were grown in SD (+Glu) medium or SD-C (−Glu) medium, respectively. **c** Box plots showing the effect of glucose starvation on Glc7-FLAG occupancy at 39 *ATG* genes. **d**, **e** ChIP-qPCR analysis of the occupancy of Glc7-FLAG (**d**) and H3pT11/H3 (**e**) at *ATG* genes when cells were grown in SD (+Glu) medium or SD-C (−Glu) medium. **f** ChIP-qPCR analysis of the occupancy of H3pT11/H3 at *ATG* genes in WT and *glc7-12* mutant when grown in SD (+Glu) medium or SD-C (−Glu) medium at 37 °C for 2 h. **g** Analysis of *ATG* gene transcription in WT, *glc7-12*, H3T11A and *glc7-12* H3T11A mutants when grown in SD (+Glu) medium or SD-C (−Glu) medium at 37 °C for 2 h by qRT-PCR. **h**, **i** ChIP-seq analysis of the binding of Glc7-FLAG at subtelomere regions when cells were grown in SD (+Glu) medium and SD-C (−Glu) medium, respectively. **j** Analysis of the transcription of telomere-proximal genes in WT, *glc7-12*, H3T11A and *glc7-12* H3T11A mutants when grown in SD (+Glu) medium or SD-C (−Glu) medium at 37 °C for 2 h by qRT-PCR. For **a**, **d**–**g**, **j**, data represent the mean ± SE of three biological independent experiments. *P < 0.05, **P < 0.01, ***P < 0.001.

H3T11 phosphorylation is regulated by glucose availability: H3T11 phosphorylation is increased with elevated glucose concentrations^13,14^. We thus investigated how Glc7-catalyzed dephosphorylation of H3T11 is regulated under starvation conditions. Cells were grown in glucose-rich (SD medium) and then switched to glucose starvation (SD-C) or nitrogen starvation (SD-N) medium. The intracellular H3pT11 levels were significantly reduced and the protein level of Glc7 was significantly increased when cells were grown in SD-C but not SD-N medium (Supplementary Fig. S6a). RT-qPCR analysis revealed that glucose starvation induced the transcription of *GLC7* (Supplementary Fig. S6b). Subcellular fractionation assay revealed that more Glc7 translocated into the nucleus under glucose starvation condition (Fig. 5a), consistent with reduced H3pT11 (Supplementary Fig. S6a).

We then performed ChIP-seq for Glc7 when cells were grown in SD (glucose rich) medium and SD-C (glucose depletion) medium, respectively. The occupancy of Glc7 at gene coding regions was significantly increased when cells were shifted from SD to SD-C medium (Fig. 5b; Supplementary Fig. S6c). The occupancy of Glc7 at *ATG* genes was also significantly increased when grown in SD-C medium (Fig. 5c; Supplementary Fig. S6d), which is further confirmed by ChIP-qPCR (Fig. 5d). In line with this data, H3pT11 was significantly reduced at *ATG* genes under glucose starvation condition (Fig. 5e); however, inactivation of Glc7 in *glc7-12* mutant increased the reduced H3pT11 at *ATG* genes to normal levels (Fig. 5f). Nonetheless, we noted that the enrichment of H3pT11 at *ATG* genes in *glc7-12* mutant was reduced when cells were grown in SD-C medium compared with SD medium (Fig. 5f), which could be caused by reduced Pyk1 activity under glucose depletion conditions^14,46^. When cells were grown under glucose starvation condition, the transcription of *ATG* genes was significantly induced (Fig. 5g). Under glucose starvation condition, the transcription of *ATG* genes was significantly reduced in *glc7-12* mutant and increased in H3T11A mutant when compared with their WT counterpart (Fig. 5g). Mutation of H3T11A increased the reduced *ATG* gene transcription in *glc7-12* mutant (Fig. 5g), suggesting that Glc7/PP1 dephosphorylates H3T11 to activate autophagy-related gene transcription under glucose starvation condition.

When cells were grown in SD-C medium, the occupancy of Glc7 at subtelomere regions was significantly increased as demonstrated by ChIP-seq (Fig. 5h, i). ChIP-qPCR also confirmed the increased binding of Glc7 at telomere-proximal genes when cells were grown under glucose starvation condition (Supplementary Fig. S6e). Upon glucose withdrawal, the occupancy of H3pT11 at telomere-proximal genes was significantly reduced and inactivation of Glc7 rescued the reduced occupancy of H3pT11 (Supplementary Fig. S6f, g), indicating that under glucose starvation, the binding of Glc7 at subtelomere regions is substantially increased to dephosphorylate H3T11.

We then examined the effect of Glc7 on Sir2 protein levels when cells were grown in SD-C medium. Sir2 was degraded by autophagy during glucose starvation (Supplementary Fig. S6h). The overall Sir2 protein levels were significantly higher in *glc7-12* mutant than WT upon glucose starvation (Supplementary Fig. S6h). ChIP-qPCR also confirmed the reduced binding of Sir2 at telomere-proximal genes when cells were grown in SD-C, while inactivation of Glc7 rescued the reduced occupancy of Sir2 (Supplementary Fig. S6i). We also examined telomere silencing in WT, *glc7-12*, H3T11A, and *glc7-12* H3T11A mutants when cells were grown under glucose starvation condition. The transcription of telomere-proximal genes was significantly increased in WT when cells were grown in SD-C medium (Fig. 5j). Under the same condition, the transcription of telomere-proximal genes was significantly reduced in *glc7-12* mutant when compared with WT (Fig. 5j). Moreover, mutation of H3T11A mutant further reduced transcription of telomere-proximal genes in *glc7-12* mutant (Fig. 5j). These data suggest that under glucose starvation, more Glc7 is expressed and translocated into the nucleus, where it binds at subtelomere regions to dephosphorylate H3T11 and reduce telomere silencing.

### Sen1 promotes Glc7-catalyzed H3T11 dephosphorylation to induce autophagy upon glucose starvation

**Fig. 6:**
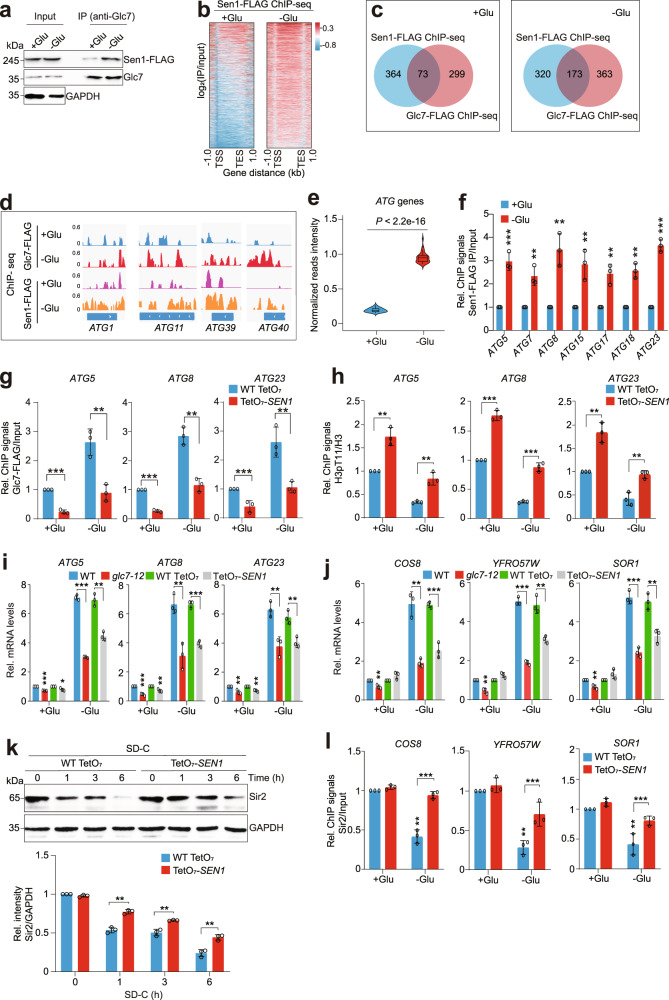
Glc7-Sen1 dephosphorylates histone H3T11 to induce autophagy-related gene transcription and reduce telomere silencing under glucose starvation conditions. **a** Co-IP showing that the interaction between endogenous Glc7 and Sen1 was increased when cells were grown in SD-C (−Glu) medium for 2 h when compared with cells grown in SD (+Glu) medium. **b** Distribution of Sen1-FLAG occupancy across each gene through 1 kb upstream of the TSS to 1 kb downstream from the TES when cells were grown in SD (+Glu) medium and SD-C (−Glu) medium, respectively. **c** Venn diagram showing the overlap of genes co-bound by Glc7-FLAG and Sen1-FLAG when cells were grown in SD (+Glu) medium or SD-C (−Glu) medium as determined by ChIP-seq. **d** ChIP-seq tracks showing the occupancy of Glc7-FLAG and Sen1-FLAG at representative *ATG* genes when cells were grown in SD (+Glu) medium or SD-C (−Glu) medium. **e** Box plots analysis of the effect of glucose starvation on the occupancy of Sen1-FLAG at 39 *ATG* genes. **f** ChIP-qPCR analysis of the occupancy of Sen1-FLAG at indicated *ATG* genes when cells were grown in SD (+Glu) medium and SD-C (−Glu) medium, respectively. **g**, **h** ChIP-qPCR analysis of the occupancy of Glc7-FLAG (**g**) and H3pT11/H3 (**h**) at *ATG* genes in WT TetO_7_ and TetO_7_-*SEN1* mutant when cells were grown in SD (+Glu) medium or SD-C (−Glu) medium supplemented with 40 μg/mL doxycycline for 2 h. **i** RT-qPCR analysis of *ATG* gene transcription in WT, *glc7-12*, WT TetO_7_ and TetO_7_-*SEN1* mutants when grown in SD (+Glu) medium or SD-C (−Glu) medium. WT and *glc7-12* mutant were grown in SD (+Glu) medium or SD-C (−Glu) medium at 37 °C for 2 h. WT TetO_7_ and TetO_7_-*SEN1* mutant were grown in SD (+Glu) medium or SD-C (−Glu) medium supplemented with 40 μg/mL doxycycline for 2 h. **j** RT-qPCR analysis of the transcription of telomere-proximal genes in WT, *glc7-12*, WT TetO_7_ and TetO_7_-*SEN1* mutants when grown in SD (+Glu) medium or SD-C (−Glu) medium. **k** Immunoblots of Sir2 in WT TetO_7_ and TetO_7_-*SEN1* mutant when grown in SD-C medium supplemented with 40 μg/mL doxycycline for 0–6 h. **l** ChIP-qPCR analysis of Sir2 occupancy at telomere-proximal genes in WT TetO_7_ and TetO_7_-*SEN1* mutant when grown in SD (+Glu) medium or SD-C (−Glu) medium supplemented with 40 μg/mL doxycycline for 2 h. For **f**–**l**, data represent the mean ± SE of three biological independent experiments. *P < 0.05, **P < 0.01, ***P < 0.001.

We next examined the effect of Glc7–Sen1 subcomplex on autophagy when cells were grown under glucose starvation condition. Similar to Glc7, the protein levels of Sen1 were significantly increased upon glucose withdrawal (Supplementary Fig. S7a). The interaction between endogenous Sen1 and Glc7 was increased upon glucose starvation (Fig. 6a). We then performed ChIP-seq for Sen1 when cells were grown in SD (glucose rich) and SD-C (glucose depletion) medium, respectively. Similar to Glc7, the occupancy of Sen1 at gene coding regions was significantly increased when cells were grown in SD-C medium when compared to those grown in SD medium (Fig. 6b). When cells were grown in SD medium, Sen1 and Glc7 co-occupied 73 genes; however, when cells were grown in SD-C medium, the number of genes co-occupied by Sen1 and Glc7 increased to 173 (Fig. 6c), which is consistent with the enhanced interaction between Sen1 and Glc7 (Fig. 6a).

The occupancy of Sen1 at *ATG* genes was significantly increased when cells were grown in SD-C medium (Fig. 6d, e), which was further confirmed by ChIP-qPCR (Fig. 6f). Upon glucose withdrawal, the increased occupancy of Glc7 at *ATG* genes was significantly reduced in TetO_7_-*SEN1* mutant (Fig. 6g), suggesting that Sen1 is also required for Glc7 binding at *ATG* genes upon glucose starvation. Under the same condition, the occupancy of H3pT11 at *ATG* genes was significantly decreased, while knockdown of *SEN1* in TetO_7_-*SEN1* mutant rescued the reduced H3pT11 at *ATG* genes (Fig. 6h). Accordingly, knockdown of *SEN1* in TetO_7_-*SEN1* mutant significantly reduced the transcription of *ATG* genes when cells were grown in SD-C medium (Fig. 6i). These data suggest that under glucose starvation, the binding of Sen1 at autophagy-related genes is increased, which recruits more Glc7 to dephosphorylate H3T11 at autophagy-related genes, leading to their transcriptional induction.

Intriguingly, although knockdown of *SEN1* had no significant effect on telomere silencing under glucose rich condition, the transcription of telomere-proximal genes was significantly reduced in TetO_7_-*SEN1* mutant upon glucose starvation (Fig. 6j), suggesting that Sen1 reduces telomere silencing only when cells were grown under glucose starvation condition. Knockdown of *SEN1* had no significant effect on H3pT11 occupancy at telomere-proximal genes upon glucose starvation (Supplementary Fig. S7b), suggesting that unlike Glc7, Sen1 reduces telomere silencing not by decreasing H3pT11 at telomere-proximal genes. Given the fact that Sen1 activates autophagy and Sir2 can be degraded by autophagy (Fig. 3f, g; Supplementary Fig. S3i), we hypothesized that Sen1 may reduce telomere silencing by promoting autophagy-mediated Sir2 degradation upon glucose starvation. To test this, we examined Sir2 protein levels in WT TetO_7_ and TetO_7_-*SEN1* mutant when cells were grown in SD-C medium. Upon glucose depletion, although the Sir2 protein levels were significantly reduced in both WT TetO_7_ and TetO_7_-*SEN1* mutant, the TetO_7_-*SEN1* mutant had significantly higher Sir2 protein levels than WT TetO_7_ (Fig. 6k), suggesting that knockdown of Sen1 represses autophagy, which may inhibit autophagy-mediated Sir2 degradation. We also examined the occupancy of Sir2 at telomere-proximal genes in WT TetO_7_ and TetO_7_-*SEN1* mutant when grown in SD and SD-C medium, respectively. The occupancy of Sir2 at telomere-proximal genes was significantly higher in TetO_7_-*SEN1* mutant than WT TetO_7_ when grown in SD-C medium despite no significant difference in SD medium (Fig. 6l), which may account for reduced transcription of telomere-proximal genes in TetO_7_-*SEN1* mutant. These results suggest that Sen1 indirectly reduces telomere silencing by promoting autophagy-mediated Sir2 degradation under glucose starvation condition.

### Rif1 facilitates Glc7-catalyzed H3T11 dephosphorylation to reduce telomere silencing upon glucose starvation

We next examined the effect of Glc7–Rif1–Rap1 complex on autophagy and telomere silencing upon glucose starvation. When cells were grown in SD-C medium, the protein levels of Rif1 remained unchanged and the interaction between Rif1 and Glc7 was unaffected (Supplementary Fig. S7a, c), which is unlike Sen1. Loss of Rif1 had no significant effect on autophagy activity upon glucose starvation (Supplementary Fig. S7d). The transcription of telomere-proximal genes was significantly reduced in *rif1Δ* mutant when cells were grown in SD-C medium (Supplementary Fig. S7e), suggesting that Rif1 derepresses the transcription of telomere-proximal genes under glucose starvation. Unlike Sen1, loss of Rif1 had no significant effect on Sir2 protein levels when cells were grown in SD-C medium (Supplementary Fig. S7f), suggesting that Rif1–Glc7 dephosphorylates H3T11 to decrease the binding of Sir2 at telomere regions instead of regulating Sir2 proteostasis to reduce telomere silencing.

Together, these results demonstrate that under glucose starvation, the transcription of *GLC7* and *SEN1* is up-regulated and more Glc7 translocates into the nucleus, where it forms the Glc7–Sen1 and Glc7–Rif1–Rap1 complexes to dephosphorylate H3T11 at autophagy-related genes and subtelomere regions, respectively. As a consequence, the transcription of autophagy-related genes is increased and telomere silencing is reduced. Moreover, the Glc7–Sen1 complex accelerates autophagy-mediated Sir2 degradation, which further reduces Sir2 binding at telomere regions and compromises telomere silencing (Supplementary Fig. S8).

### PPP1CA–SETX dephosphorylates H3T11 to regulate autophagy in mammals

**Fig. 7:**
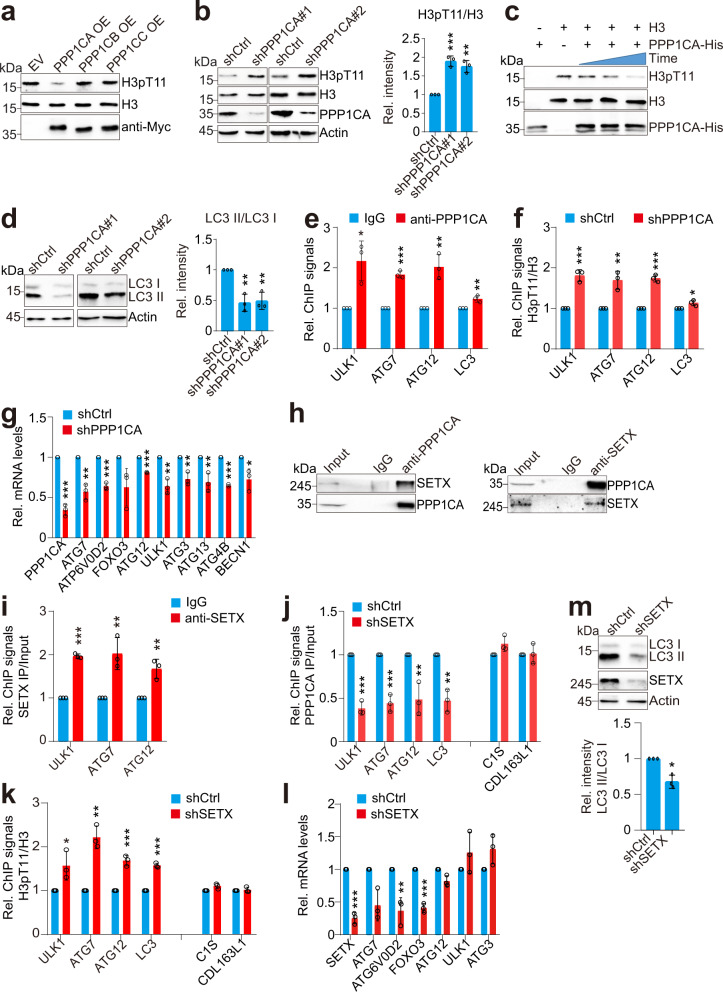
PPP1CA–SETX dephosphorylates histone H3T11 to regulate autophagy in mammals. **a** Immunoblot analysis of H3pT11 in HeLa cells transfected with plasmids overexpressing PPP1CA, PPP1CB and PPP1CC. **b** Immunoblot analysis of H3pT11 in control (shCtrl) and PPP1CA-knockdown (*shPPP1CA*#1, *shPPP1CA*#2) HeLa cells. **c** The purified recombinant PPP1CA dephosphorylates H3T11 as determined by in vitro dephosphorylation assay. **d** Analysis of autophagy activity in control (shCtrl) and PPP1CA-knockdown (*shPPP1CA*#1, *shPPP1CA*#2) HeLa cells. **e** ChIP-qPCR analysis of PPP1CA occupancy at autophagy-related genes (*ULK1*, *ATG7*, *ATG12*, *LC3*) in HeLa cells. **f** ChIP-qPCR analysis of H3pT11/H3 occupancy at autophagy-related genes in control (shCtrl) and PPP1CA-knockdown (*shPPP1CA*) HeLa cells. **g** RT-qPCR analysis of the transcription of autophagy-related genes in shCtrl and *shPPP1CA* HeLa cells. **h** Co-IP and reciprocal IP showing the interaction between PPP1CA and SETX. **i** ChIP-qPCR analysis of SETX occupancy at autophagy-related genes in HeLa cells. **j** ChIP-qPCR analysis of PPP1CA occupancy at autophagy-related genes in control (shCtrl) and SETX-knockdown (*shSETX*) HeLa cells. **k** ChIP-qPCR analysis of H3pT11/H3 occupancy at autophagy-related genes in shCtrl and *shSETX* HeLa cells. The telomere-proximal genes, *C1S* and *CDL163L1* were used as controls. **l** RT-qPCR analysis of the transcription of autophagy-related genes in shCtrl and *shSETX* HeLa cells. **m** Analysis of autophagy activity in shCtrl and *shSETX* HeLa cells. For **b**, **d**–**g**, **i**–**m**, data represent the mean ± SE of three biological independent experiments. *P < 0.05, **P < 0.01, ***P < 0.001.

Next, we asked whether Glc7/PP1-mediated dephosphorylation of H3T11 is conserved in mammals. We first treated HeLa cells with PP1 inhibitor, tautomycetin^47^. The intracellular H3pT11 was significantly increased by tautomycetin in a dose-dependent manner (Supplementary Fig. S9a). We then individually overexpressed PPP1CA (PP1 catalytic subunit, α isoform), PPP1CB (PP1 catalytic subunit, β isoform), and PPP1CC (PP1 catalytic subunit, γ isoform)^48^ in HeLa cells and found that only overexpression of PPP1CA markedly reduced the global H3pT11 (Fig. 7a). To confirm that, we designed two independent shRNAs (shPPP1CA) to knockdown the expression of PPP1CA in HeLa cells. H3pT11 was significantly increased in PPP1CA-knockdown cells (Fig. 7b). Furthermore, we purified the recombinant PPP1CA and performed in vitro dephosphorylation assay with recombinant histone H3 pre-phosphorylated at H3T11. PPP1CA directly dephosphorylated H3T11 in a time-dependent manner (Fig. 7c). These results indicate that PPP1CA is the primary phosphatase that dephosphorylates H3T11 in mammals.

To determine whether PPP1CA regulates autophagy, we analyzed the conversion of non-lipidated LC3-I to lipidated LC3-II, as a common marker of autophagy activity in control (shCtrl) and PPP1CA-knockdown (*shPPP1CA*) cells. Knockdown of PPP1CA significantly reduced LC3-II as well as the ratio of LC3-II/LC3-I in HeLa and primary human vein endothelial cells (HUVECs) cells (Fig. 7d; Supplementary Fig. S9b), suggesting that similar to PP1/Glc7, PPP1CA activates autophagy in mammals. To determine whether PPP1CA regulates autophagy by dephosphorylating H3T11, we performed ChIP-qPCR for PPP1CA and our results revealed that PPP1CA directly bound at autophagy-related genes (*ULK1*, *ATG7*, *ATG12*, *LC3*) and knockdown of PPP1CA significantly increased the enrichment of H3pT11 at autophagy-related genes (Fig. 7e, f). In addition, knockdown of PPP1CA significantly reduced the transcription of autophagy-related genes (Fig. 7g), suggesting that PPP1CA dephosphorylates H3T11 at autophagy-related genes to activate their transcription.

Next, we examined whether PPP1CA interacts with the homolog of Sen1, senataxin (SETX) in mammalian cells. The Co-IP and reciprocal IP assay confirmed the interaction between PPP1CA and SETX (Fig. 7h). ChIP-qPCR analysis revealed significant occupancy of SETX at the autophagy-related genes (Fig. 7i). Depletion of SETX significantly reduced the binding of PPP1CA and increased the occupancy of H3pT11 at autophagy-related genes (Fig. 7j, k). In addition, knockdown of SETX significantly reduced the transcription of autophagy-related genes (Fig. 7l). Knockdown of SETX significantly reduced autophagy activity as indicated by reduced LC3-II and the ratio of LC3-II/LC3-I (Fig. 7m). All these data suggest that PPP1CA–SETX dephosphorylates H3T11 and transcriptionally activates autophagy in mammals.

### PPP1CA–Rif1 dephosphorylates H3T11 to regulate telomere silencing and cellular senescence in mammals

**Fig. 8:**
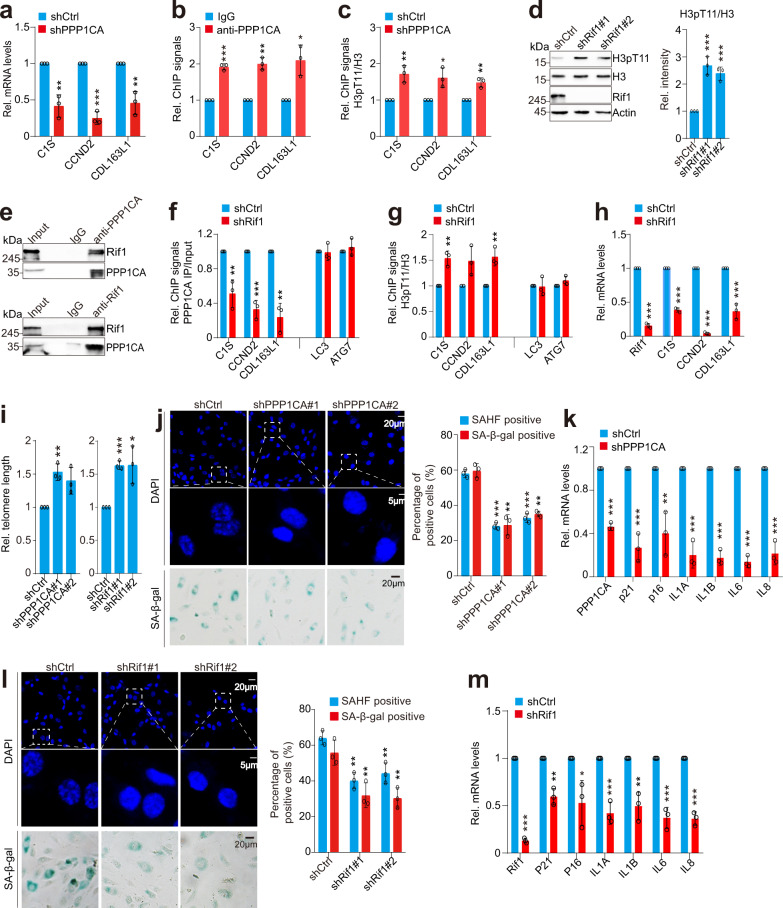
PPP1CA–Rif1 dephosphorylates H3T11 to regulate telomere silencing and cellular senescence in mammals. **a** RT-qPCR analysis of the transcription of telomere-proximal genes (*C1S*, *CCND2*, *CDL163L1*) in control (shCtrl) and PPP1CA-knockdown (*shPPP1CA*) HUVECs. **b** ChIP-qPCR analysis of PPP1CA occupancy at telomere-proximal genes in HUVECs. **c** ChIP-qPCR analysis of the occupancy of H3pT11/H3 at telomere-proximal genes in control (shCtrl) and PPP1CA-knockdown (*shPPP1CA*) HUVECs. **d** Immunoblot analysis of H3pT11 in control (shCtrl) and Rif1-knockdown (*shRif1*#1, *shRif1*#2) HUVECs. **e** Co-IP and reciprocal IP showing the interaction between PPP1CA and Rif1. **f** ChIP-qPCR analysis of PPP1CA occupancy at telomere-proximal genes in control (shCtrl) and Rif1-knockdown (*shRif1*) HUVECs. **g** ChIP-qPCR analysis of H3pT11/H3 occupancy at telomere-proximal genes in control (shCtrl) and Rif1-knockdown (*shRif1*) HUVECs. **h** RT-qPCR analysis of the transcription of telomere-proximal genes in control (shCtrl) and Rif1-knockdown (*shRif1*) HUVECs. **i** Knockdown of PPP1CA and Rif1 significantly increased telomere length. **j** Effect of PPP1CA knockdown on the senescence of HUVECs as determined by SAHF formation (DAPI staining) and SA-β-gal staining. Right panel: quantification of the number of SAHF-positive and SA-β-gal positive HUVECs. **k** RT-qPCR analysis of the transcription of *P21*, *P16*, *IL1A*, *IL1B*, *IL6* and *IL8* in shCtrl and *shPPP1CA* HUVECs. **l** Effect of Rif1 knockdown on the senescence of HUVECs as determined by SAHF formation (DAPI staining) and SA-β-gal staining. **m** RT-qPCR analysis of the transcription of *P21*, *P16*, *IL1A*, *IL1B*, *IL6* and *IL8* in shCtrl and *shRif1* HUVECs. For **a**–**d**, **f**–**m**, data represent the mean ± SE of three biological independent experiments. *P < 0.05, **P < 0.01, ***P < 0.001.

We then examined whether PPP1CA regulates the transcription of telomere-proximal genes in mammals. CCDN2, C1S and CDL163L1, which are located 0–10 Mb from the end of chromosome 12p, were used to determine the telomere position effect in mammals^49^. Knockdown of PPP1CA significantly reduced the transcription of these three genes in HUVECs (Fig. 8a). ChIP-qPCR analysis revealed that PPP1CA directly bound these three genes and knockdown of PPP1CA significantly increased the occupancy of H3pT11 at these genes (Fig. 8b, c), suggesting that PPP1CA dephosphorylates H3T11 to impact on telomere silencing in mammals.

Knockdown of SETX had no significant effect on PPP1CA binding and H3pT11 occupancy at telomere-proximal genes (Fig. 7j, k). We then examined whether the homolog of Rif1 recruits PPP1CA to dephosphorylate H3T11 at subtelomere regions. Indeed, knockdown of Rif1 significantly increased the intracellular H3pT11 (Fig. 8d). PPP1CA interacted with Rif1 as determined by Co-IP and reciprocal IP (Fig. 8e). In addition, knockdown of Rif1 significantly reduced the binding of PPP1CA and increased the occupancy of H3pT11 at telomere-proximal genes (Fig. 8f, g). Knockdown of Rif1 significantly reduced the transcription of these telomere-proximal genes (Fig. 8h). The transcription of telomere-proximal genes is regulated by telomere length^49^. We thus examined the effect of PPP1CA/Rif1 on telomere length in HUVECs. Knockdown of PPP1CA and Rif1 significantly increased the telomere length (Fig. 8i), consistent with their effects on telomere silencing.

As telomere length is closely associated with cellular senescence, we thus examined the effect of PPP1CA–Rif1 on cellular senescence by analyzing senescence-associated β-galactosidase (SA-β-gal) staining, senescence-associated heterochromatin foci (SAHF) formation as visualized by DAPI staining^50,51^. Knockdown of PPP1CA significantly reduced SA-β-gal staining and SAHF formation in HUVECs (Fig. 8j). Moreover, knockdown of PPP1CA significantly reduced the expression of p21 and proinflammatory cytokines (IL1A, IL1B, IL6, IL8) associated with the senescence-associated secretory phenotype (SASP) (Fig. 8k), indicating that PPP1CA accelerates the early onset of cellular senescence. Similarly, knockdown of Rif1 also significantly reduced premature cellular senescence as indicated by reduced SA-β-gal staining and SAHF formation as well as decreased expression of p21 and SASP genes (Fig. 8l, m; Supplementary Fig. S9c).

Collectively, these data suggest that PPP1CA–Rif1 dephosphorylates H3T11 at subtelomere regions, which in turn impacts telomere silencing, telomere length and cellular senescence.

## Discussion

Cells need to coordinate gene expression with their metabolic states but the underlying mechanisms is not well understood. Pyruvate kinase phosphorylates histone H3T11 to repress autophagy and maintain telomere heterochromatin under nutrient-rich condition^14,15^. However, it is poorly understood how H3T11 phosphorylation is removed and dynamically regulated in response to nutritional changes. In this work, we establish Glc7/PP1 as the enzyme that dephosphorylates H3T11 and identify two conserved Glc7-containing complexes as regulators of autophagy and telomere structure. When glucose is depleted, the expression of Glc7 is up-regulated, which then translocates into the nucleus to dephosphorylate H3T11 in the form of Glc7–Sen1 and Glc7–Rif1–Rap1 complexes. The Glc7–Sen1 complex dephosphorylates H3T11 to transcriptionally activate autophagy. The Glc7–Rif1–Rap1 complex dephosphorylates H3T11 to reduce telomere silencing. Interestingly, the Glc7–Sen1 complex accelerates autophagy-mediated degradation of Sir2, leading to reduced telomere silencing. Most importantly, both Glc7–Sen1 and Glc7–Rif1 complexes are conserved in mammals. PPP1CA interacts with SETX to dephosphorylate H3T11 and transcriptionally activate autophagy. PPP1CA interacts with Rif1 to dephosphorylate H3T11 at telomere-proximal regions, which then reduces telomere silencing, decreases telomere length and accelerates cellular senescence. Our results thus uncover a conserved mechanism by which PP1 dephosphorylates H3T11 to regulate gene expression and chromatin structure in response to nutrient availability.

PP1 is a ubiquitous eukaryotic enzyme that regulates a variety of cellular processes by catalyzing the dephosphorylation of its substrates^52^. The eukaryotic organisms contain multiple genes encoding PP1 with the exception for budding yeast, which has a single gene, *GLC7*, that encodes PP1^53^. This makes the yeast Glc7 as a good example to study the function and regulation of PP1. In this work, we identify histone H3T11 as the new target for Glc7/PP1 and reveal its role in transcriptional activation of autophagy. The genome-wide binding pattern for Glc7/PP1 anti-correlates that of H3T11 phosphorylation. As Pyk1 also binds at the coding regions in addition to gene promoter regions (Supplementary Fig. S1e), the preferential binding of Glc7 at gene coding regions partially explains why H3T11 phosphorylation is absent at gene coding regions. Another finding of this work is that the expression and nuclear localization of Glc7/PP1 are regulated in response to glucose availability. Much is known about regulation of Glc7/PP1 activity by other protein factors, i.e. Glc8 is a major activator of Glc7/PP1 activity, while proline isomerases Fpr3 and Fpr4 are Glc7/PP1 inhibitors^54,55^. We find that Glc7/PP1 can be regulated at the transcriptional level. Combined with the Pyk1 study^15^, our data support a model in which Pyk1 and Glc7 antagonistically control H3T11 phosphorylation to regulate autophagy in response to nutrient availability. When glucose is abundant, Pyk1 phosphorylates H3T11 to repress autophagy, which maintains autophagy at low basal level. However, when glucose is exhausted, the expression of Glc7 and Sen1 is up-regulated. More Glc7 translocates into the nucleus and the interaction between Glc7 and Sen1 is enhanced, which promotes the dephosphorylation of H3T11 at autophagy-related genes, leading to induction of autophagy. Therefore, our work not only provides the first evidence that Glc7/PP1 transcriptionally activates autophagy, but also provides a direct epigenetic mechanism by which autophagy is induced upon glucose starvation.

Wang et al. reported that in normal hepatocytes, fructose-1,6-bisphosphatase 1 (Fbp1) can translocate into the nucleus to dephosphorylate histone H3T11^56^. We thus examined the effect of *FBP1* deletion on H3T11 phosphorylation in budding yeast. However, there was no significant change of intracellular H3pT11 between WT and *fbp1Δ* mutant (Supplementary Fig. S10a). Moreover, deletion of *FBP1* had no significant effect on the transcription of autophagy-related genes as well as telomere-proximal genes (Supplementary Fig. S10b–d). We also constructed *fbp1Δ* and *glc7-12 fbp1Δ* mutants. Immunoblot analysis showed that there was no significant difference between *glc7-12* and *glc7-12 fbp1Δ* mutants (Supplementary Fig. S10e). We also examined the effect of Fbp1 on H3pT11 occupancy at autophagy-related genes (*ATG*) and telomere-proximal genes. Our data showed that loss of Fbp1 had no significant effect on H3pT11 occupancy at these genes (Supplementary Fig. S10f). There was also no significant difference for H3pT11 in *glc7-12* and *glc7-12 fbp1Δ* mutants (Supplementary Fig. S10f). These data suggest that yeast cells use Glc7/PP1 instead of Fbp1 to dephosphorylate H3T11.

Glc7 interacts with a multitude of regulators to control a diversity of processes such as glycogen metabolism, vesicle trafficking, cell polarity, DNA damage, gene transcription, and cell-cycle progression^27^. The extensively studied Glc7 regulator is Reg1. Glc7–Reg1 dephosphorylates and deactivates Snf1 in the cytoplasm when glucose is abundant^57^. In addition, Glc7–Reg1 dephosphorylates and inactivates enzymes unnecessary for growth on glucose, such as Hxk2^58^. However, loss of Reg1 has no effect on Glc7-mediated H3T11 dephosphorylation. Glc7 has been reported to be a subunit of the cleavage and polyadenylation factor (CPF) with Sen1, Nrd1 and Nab3 in the nucleus^59^. Loss of Sen1 significantly reduces the binding of Glc7 at autophagy-related genes, which increases H3T11 phosphorylation at autophagy-related genes. However, knockdown of Nrd1 and Nab3 has no significant effect on H3T11 phosphorylation, suggesting that Sen1 but not CPF complex is involved in recruitment of Glc7 to autophagy-related genes. Thus, Glc7–Sen1 could be a new complex that dephosphorylates H3T11 to activate autophagy. Although we cannot exclude the possibility that Glc7–Sen1 may directly regulate the transcription of autophagy-related genes, Glc7–Sen1-mediated H3T11 dephosphorylation could be a primary mechanism. As knockdown of Sen1 significantly increased the global H3pT11 level, the Glc7–Sen1 complex may not only dephosphorylate H3T11 at autophagy-related genes but also other genes. Indeed, by analyzing the ChIP-seq data for Sen1 and Glc7, we found that they co-localized at 73 genes, which are enriched in RNA degradation, fatty acid metabolism, autophagy, glycolysis pathways, and etc.

We identify a conserved role of Sen1 in regulating autophagy. Sen1/SETX recruits Glc7/PPP1CA to autophagy-related genes to dephosphorylate H3T11, which then activates the transcription of autophagy-related genes. Richard et al. reported that knockdown of SETX reduces autophagy flux but how SEXT regulates autophagy remains unknown^60^. Our work provides a possible mechanism by which SETX activates autophagy by facilitating Glc7/PPP1CA-mediated H3T11 dephosphorylation. Interestingly, although Sen1 has no significant effect on telomere silencing under normal condition, Sen1 reduces telomere silencing by promoting Sir2 degradation under glucose starvation. This could be explained by the fact that under normal conditions, autophagy activity is low and H3T11 phosphorylation at subtelomere regions is relatively high, which restricts the nucleus export of Sir2 and protects Sir2 from being degraded by autophagy. Upon glucose starvation, autophagy activity is enhanced and H3T11 phosphorylation at subtelomere regions is reduced. Sir2 is dissociated from subtelomere regions, and translocates into the cytoplasm followed by degradation by autophagy. Under this condition, knockdown of Sen1 decreases autophagy activity, which may reduce autophagy-mediated Sir2 degradation and enhances telomere silencing.

Rif1 has been reported to act as a Glc7 substrate-targeting subunit to prevent premature DNA replication origin firing by directing Glc7-catalyzed dephosphorylation of Minichromosome Maintenance complex^61^. Glc7 also acts as a Rif1 effector to suppress the double stranded break resection^62^. Sen1 has been reported to contain a “RVXF” Glc7 interaction motif (K_1999_SVAF_2003_) and mutation of F2003A abolished the interaction between Glc7 and Sen1^59^. By analyzing the sequence of Rif1, we also identified a similar “RVXF” motif, K_114_SVAF_118._ It is possible that this “RVXF” motif is required for the interaction between Rif1 and Glc7. Moreover, the interaction between Rif1 with Glc7 is essential for suppression of TG repeat elongation^28^. Artificially tethered Glc7 can bypass the role of Rif1 in suppressing telomere extension^28^. However, it remains unknown about the target protein(s) of Glc7–Rif1^28^. Here, we find that Rap1 recruits Rif1 to subtelomere regions, which then targets Glc7 to dephosphorylate histone H3T11 at subtelomere regions. Loss of Glc7–Rif1–Rap1 subcomplex increased H3T11 phosphorylation at subtelomere regions to enhance telomere silencing. Most importantly, the functions of Glc7 and Rif1 are conserved in mammalian cells: Rif1 directs PPP1CA to dephosphorylate H3T11 at subtelomere regions, which in turn reduces telomere silencing, shortens telomere length and accelerates cellular senescence. As deletion of *RIF1* significantly increased the global H3T11 phosphorylation level, the Glc7–Rif1–Rap1 complex may not only dephosphorylate H3T11 at subtelomere regions. Like Glc7–Sen1 complex, the Glc7–Rif1–Rap1 complex may dephosphorylate H3T11 at other euchromatin regions enriched with Rif1.

Although Rap1 recruits both Rif1 and Rif2 to telomere regions, only Rif1 affects Glc7-catalyzed H3T11 dephosphorylation. Loss of Rif2 has no significant effect on H3T11 phosphorylation. This differential behavior of Rif1 and Rif2 on telomere structure is consistent with the report by the Donaldson group, despite little is known about the underlying mechanism^28^. Loss of Rif1 but not Rif2 increases telomere silencing^40,63^. Although both Rif1 and Rif2 can limit telomere elongation, Rif1 and Rif2 appear to act through different pathways as they have additive effects on telomere length^40,41^. One plausible explanation is that Rif1 and Rif2 may interact with different proteins once recruited to telomeres by Rap1^40^. In our case, Rif1 but not Rif2 interacts with Glc7. It has been proposed that Rif1 regulate telomere length in part by competing with the Sir2/Sir3/Sir4 complex for binding to the C-terminus of Rap1^40^. It remains unclear how Rif1 competes with the Sir2/Sir3/Sir4 complex. As H3T11 phosphorylation promotes the binding of Sir2/Sir3/Sir4 complex at subtelomere regions^15^, it is possible that Rif1 recruits Glc7 to dephosphorylate H3T11, which then dissociates the Sir2/Sir3/Sir4 complex at subtelomere regions. Our study hence provides a possible explanation about how Rif1 competes the Sir2/Sir3/Sir4 complex to bind Rap1.

PPP1CA is a PPP1C isoform that contributes to the senescence program induced by oncogenic Ras^64^. Oncogenic Ras promotes an increase in the intracellular levels of ceramides together with an increase in the PPP1CA protein level, which maintains pRb in a hypophosphorylation status^64^. Here, we report that knockdown of PPP1CA significantly reduced replicative senescence and uncovered a new mechanism by which PPP1CA promotes cellular senescence. PPP1CA dephosphorylates H3T11 at subtelomere regions, which reduces telomere silencing and shortens telomere length. Interestingly, a more recent study reported that PPP1CA is subjected to telomere silencing over long distance^24^. In senescent cells, PPP1CA expression is up-regulated, which could form a feedback loop to reduce telomere silencing and accelerate cellular senescence^24^. By directing PPP1CA to dephosphorylate H3T11 at subtelomere regions, Rif1 reduces telomere length and accelerates cellular senescence. Due to technical issues, it is difficult to obtain SETX-knockdown HUVECs and assess the effect of SETX on cellular senescence. Nonetheless, our study suggests that PPP1CA and Rif1 are potential targets for intervening premature cellular senescence.

In summary, we establish Glc7/PP1 as the enzyme that specifically dephosphorylates histone H3T11. Moreover, we identify two conserved PP1/substrate-targeting subcomplexes that regulate autophagy and telomere silencing. Our study uncovers a conserved mechanism to dynamically regulate H3T11 phosphorylation and reveals an epigenetic mechanism to regulate autophagy and telomere structure in response to nutrient availability.

## Materials and methods

### Yeast strains and cell lines

All yeast strains used in this study were listed in Supplementary Table S1. The gene deletion mutants and genomic integration of C-terminal epitope tags were constructed by standard protocols. All yeast strains were verified by colony PCR, DNA sequencing, RT-qPCR, and/or immunoblots before being used for experiments.

The primary HUVECs were purchased from ScienCell Research Laboratories. Cells were grown in Endothelial Cell Medium supplemented with 5% of FBS, 1% of Endothelial Cell Growth Supplement and 1% of penicillin/streptomycin solution. The HeLa and HEK293T cells were obtained from the American Type Culture Collection. The HeLa and HEK293T cells were maintained in Dulbecco’s modified Eagle’s medium supplemented with 10% FBS and 1% of penicillin*/*streptomycin solution. All cell lines used in this study were reauthenticated by short tandem repeat analysis after resuscitation in our laboratory.

### Cell growth and treatment

Yeast cells were grown in 2% glucose-containing YPD (Yeast Extract Peptone Dextrose) medium at 30 °C until OD_600_ of 0.7–1.0. For *glc7-12* mutant, cells were grown in 26 °C until OD_600_ of 0.5 and then transferred to pre-warmed medium at 37 °C for 2 h. For conditional knockdown mutants (WT TetO_7_, TetO_7_-*SEN1*, TetO_7_-*CDC14*, TetO_7_-*SSU72*, TetO_7_-*NRD1*, TetO_7_-*NAB3*, TetO_7_-*CDC13*, TetO_7_-*RAP1*, TetO_7_-*TEN1*, TetO_7_-*RFA2*, TetO_7_-*YPI1*, TetO_7_-*SDS22*), cells were grown in YPD medium at 30 °C until OD_600_ of 0.5–0.7 and then treated with 40 μg/mL doxycycline for 2 h. Due to the lack of commercial antibodies against these proteins, the knockdown efficiency was examined by RT-qPCR. The knockdown efficiency results were presented in Supplementary Figs. S4b and S11a. For nitrogen starvation treatment, cells were grown in SD medium (0.67% yeast nitrogen base without amino acids, supplemented with amino acids and 2% glucose) until OD_600_ of 0.7 and then pelleted at 3000 rpm for 5 min. After being washed with sterile ddH_2_O, cells were grown in SD-N medium (0.17% yeast nitrogen base without amino acids and ammonium sulfate, 2% glucose, additional amino acids that are required for the growth of an auxotrophic strain) for 0–6 h. For glucose starvation treatment, cells were grown in YPD until OD_600_ of 0.7 and then pelleted at 3000 rpm for 5 min. After being washed with sterile ddH_2_O, cells were grown in SD-C medium (0.67% yeast nitrogen base without amino acids, supplemented with amino acids) for 0–6 h. For rapamycin treatment, cells were grown in YPD until OD_600_ of 0.7. 2 μg/mL rapamycin was added into the medium and cells were then grown for 0–1.5 h.

### Immunoblot analysis

Cells were grown in 5 mL YPD or selective medium until OD_600_ of 0.7–1.0. Cells were then harvested and lysed in alkaline lysis buffer (2 M NaOH, 8% 2-mercaptoethanol). After centrifugation, the protein pellet was resuspended in 150 μL 2× SDS-sample buffer. Protein samples were separated by 8%–15% SDS-PAGE and transferred to PVDF membrane. The blots were probed by primary antibodies followed by incubation with horseradish peroxidase-labelled IgG secondary antibodies. The protein bands were visualized using the ECL Chemiluminescence Detection Kit (Bio-Rad, 170-5061) and quantified with Image J software (v.1.8.0).

### Antibodies

Antibodies against H3 (1: 5000; ab1791), H3pT11 (1:3000; ab5168) and SETX (1:3000; ab243904) were purchased from Abcam; antibody against FLAG M2 (1:3000; F1804-1MG) was obtained from Sigma-Aldrich; antibodies against Sir2 (1:500; sc-6667) and LC3 (1:1000; sc-398822) were purchased from Santa Cruz Biotechnology; antibodies against Myc (1:5000; 60003-2-1 g), GAPDH (1:10000; 10494-1-AP), beta-actin (1:10000; 20536-1-AP), GFP (1:5000; 66002-1-Ig) and goat polyclonal anti-mouse IgG (1:5000; SA00001-1) were obtained from Proteintech; antibody against Rif1 (1:3000; 95558 S) was purchased from Cell Signaling Technology; antibodies against CDKN1A/p21 (1:3000; A11454), and PPP1CA (1:5000; A12468) were purchased from Abclonal; antibody against CBP (1:3000; abs130593) was purchased from Absin. Antibody against Glc7 (1:5000) was custom-made in Abclonal and its specificity was confirmed by dot blots with Glc7 peptide (Supplementary Fig. S11b). H3pT11 was validated for ChIP assay in H3T11A mutant and PKM2 knockdown HUVECs (Supplementary Fig. S11c, d).

### Chromatin immunoprecipitation (ChIP) assay

For ChIP assay in yeast, cells were grown in 200 mL YPD media at 30 °C until OD_600_ of ~0.7–1.0. The crosslinking was performed in 1% formaldehyde and quenched by adding 10 mL of 2.5 M glycine. Cells were harvested, washed once with cold TBS plus PMSF, lysed in FA-SDS buffer (40 mM HEPES pH7.5, 1 mM EDTA pH8.0, 0.1% SDS, 1% Triton X-100, 0.1% Na deoxycholate, 1 mM PMSF, 2 μg/mL leupeptin, 1 μg/mL pepstatin A, protease inhibitor cocktail, phosphatase inhibitor cocktail). Chromatin was sonicated to an average size of ~500 bp and then subjected to IP with antibodies pre-bound to Protein G Dynabeads (Invitrogen) at 4 °C overnight. The beads were washed successively with FA buffer, FA buffer plus 1 M NaCl, FA buffer plus 0.5 M NaCl, TEL buffer (10 mM Tris pH 8.0, 1 mM EDTA, 0.25 M LiCl, 1% NP-40, 1% Na deoxycholate) and TE buffer (10 mM Tris pH 7.4, 1 mM EDTA). The eluted DNA/protein complex was treated with 20 µg Proteinase K at 55 °C for 1 h followed by treatment at 65 °C overnight. After removing RNA by RNase (Roche), the DNA was purified with ethanol precipitation and quantitated by qPCR with primers listed in Supplementary Table S2.

For ChIP-seq, the libraries were constructed and sequenced on an Illumina platform as described^65^. Reads were aligned to yeast genome sacCer3 from UCSC using bowtie2 version 2.1.0 with parameter -k 1. The telomere repeated sequences were excluded. Data was put into R (3.1.0) for further analysis. The binding peaks were called using MACS2 (v.2.1.1, macs2 callpeak) with parameter -t -c -g 1.2e7 -n -B -q 0.01 --nomodel. Peaks annotation was performed on a website service (https://manticore.niehs.nih.gov/pavis2/). Tracks were smoothed by deepTools2 (v.2.0) and visualized by IGV software (v.2.0) with a reference genome (sacCer3).

For mammalian cells, ChIP was performed as described^66^. In brief, cells were cross-linked with 1% formaldehyde and quenched by 0.125 M glycine. After that, cells were collected, washed and lysed in buffer B (50 mM Tris pH8.0, 5 mM EDTA, 1% SDS, 1 mM PMSF, 1:100 protease inhibitor cocktail). Chromatin was sheared by sonication and subjected to immunoprecipitation with antibodies pre-bound to Protein G Dynabeads (Invitrogen) overnight. Beads were washed and the eluted DNA*/*protein complex was then treated with 20 μg Proteinase K at 55 °C for 2 h and the crosslink was reversed at 65 °C overnight. After digestion with RNase (Roche), the DNA was purified and quantitated by qPCR with primers listed in Supplementary Table S2.

### RT-qPCR

Total RNA was extracted from yeast cells by the phenol-chloroform extraction method^15^. Total RNA was extracted from HUVEC and HeLa cells by TRIzol reagent RNAiso Plus (Takara) as described^66^. Purified RNA was digested with DNase (Sigma) and reversed transcribed into cDNA using Reverse Transcriptase Kit (M-MLV) (ZOMANBIO). The RNA quality was determined by agarose gel electrophoresis. 0.5 µg RNA was taken for cDNA synthesis in NovoScript®Plus All-in-one 1st Strand cDNA Synthesis SuperMix (Novoprotein). The qPCR was performed using iTaq™ Universal SYBR® Green Supermix (Bio-Rad, 1725121) with primers listed in Supplementary Table S2.

### RNA-seq

Total RNA was isolated from exponential growing yeast cells by standard phenol-chloroform extraction procedures and the quality of RNA was examined using Agilent Bioanalyzer according to the manufacturer’s instructions. Library construction, sequencing and bioinformatics analysis were performed by MegaGenomics Inc. (Beijing, China). The *P* value was calculated by edgeR (v3.24). The differentially expressed genes (DEGs) were defined as *P* < 0.05 and log_2_ fold change (FC) ≥ 0.75 or log_2_FC ≤ 0.75. Three biological replicates were performed for WT and *glc7-12* mutant.

Genes within 60 kb of a telomere throughout the genome were pooled and ordered according to their distance from a telomere^65^. The genes were grouped by their distance from the nearest telomeres in consecutive intervals of 10 kb. For each interval, the fraction of genes that were repressed in the mutant was calculated. A *χ*^*2*^ value for each interval was calculated by comparing the fraction of genes that were repressed in the interval to the genome-wide average. The statistical significance is defined as the following: *χ*^*2*^ > 3.841, *P* < 0.05; *χ*^*2*^ > 6.635, *P* < 0.01; *χ*^*2*^ > 10.828, *P* < 0.001.

### Microscopy analysis

The microscopy and imaging processing were performed as described^15,67^. Yeast cells were cultured in YPD medium until OD_600_ of 1.0. After washing with cold PBS, cells were fixed with 4% formaldehyde in PBS for 0.5 h and treated with DAPI (Solarbio) for 15 min at room temperature. Cells were then washed with cold PBS. The cell morphology was visualized using a ZEISS LSM710 microscope (Germany) with a 100× oil immersion objective by fluorescent microscopy. Images were acquired using ZEN Imaging Software ZEN 2.1 (ZEISS). The merged color Images were generated by ImageJ.

### Sample preparation and MS analysis

Yeast cells were grown in 1.2 L YPD media until an OD_600_ of 1.0, washed with PBS and flash frozen in liquid nitrogen. Thawed cell pellets were resuspended in IP buffer (40 mM HEPES pH7.5, 150 mM NaCl, 10% glycerol, 0.1% Tween-20) and lysed with glass beads using a Biospec bead beater. The lysate was centrifuged at 45,000 rpm at 4 °C for 1.5 h and the supernatant was incubated with anti-FLAG agarose beads at 4 °C for 4 h. The beads were washed extensively in IP buffer. Proteins were disulphide-reduced by 25 mM DTT at 37 °C for 40 min, alkylated by adding 50 mM iodoacetamide, and then digested with sequencing-grade trypsin (Promega) at 37 °C overnight. The supernatant was desalted using C18 solid-phase cartridges and lyophilized. The dried peptides were reconstituted in 0.1% FA and loaded onto an Acclaim PepMap 100 C18 LC column (Thermo Fisher) utilizing a Thermo Easy nLC 1000 LC system (Thermo Fisher) connected to Q Exactive HF mass spectrometer (Thermo Fisher) and data were analyzed as described^68^.

### Structure modeling

The structure modeling was performed as described^67,68^. The crystal structure of Glc7 was obtained from the Protein Data Bank (PDB code 1ATP). To visualize the docked conformation, the PyMol molecular graphics system was used, which also removed water molecules and peptide inhibitor PKI. The structure of H3pT11 peptide (S10pT11G12) was generated using I-TASSER protein Structure and Function Prediction web server (http://zhanglab.ccmb.med.umich.edu/I-TASSER/). Molecular docking simulation of Glc7 and H3pT11 peptide was performed with the AutoDock Vina. All residues within Glc7 binding site were included using the following spatial coordinates of the central cavity: *x* = 126, *y* = 126, and *z* = 124. The coordinates of the grid resolution were *x* = 50.018, *y* = 50.565, *z* = 46.656.

### In vitro dephosphorylation assay

5 μg purified Glc7 was incubated with 100 ng recombinant purified histone H3 or in vitro-assembled nucleosomes in 30 μL dephosphorylation buffer (50 mM HEPES pH7.5, 100 mM NaCl, 2 mM DTT, 0.01% Brij 35, 0.1 mM EGTA, 50% glycerol, 10 mM MnCl_2_) at 30 °C for 0–1 h. The reaction was quenched by adding 2× SDS-sample buffer and boiling for 5 min. The reaction products were then subjected to immunoblots with anti-H3pT11 and anti-H3 antibodies.

For in vitro dephosphorylation assay with PPP1CA, 1 μg purified PPP1CA was incubated with 100 ng recombinant purified histone H3 in 40 μL dephosphorylation buffer at 37 °C for 0–1 h.

### siRNA and shRNA transfection

Cells were transfected with siRNA using Neofect according to manufacturer’s instructions. To construct stable knockdown cell lines, shRNA hairpins were cloned into the lentiviral vector pLKO.1. A control hairpin that targets GFP was cloned into the pLKO.1 vector and used as a negative control. HEK293T cells were transfected with pLKO.1 vectors and lentiviral packaging vectors. 48 h later, the lentivirus was collected. Cells were transfected with lentivirus and selection was performed under 2 μg/mL puromycin. The knockdown efficiency was examined by RT-qPCR and immunoblots. The siRNA and shRNA sequences used in this study were listed in Supplementary Table S3.

### Senescence-associated β-galactosidase (SA-β-gal) staining

The SA-β-gal activity of HUVECs was assessed by the Senescence β-Galactosidase Staining Kit (Beyotime)^65,69^. Cells were cultured in 6-well plates, washed with PBS and fixed for 10–15 min at room temperature. Cells were then washed twice with PBS and incubated with the staining mixture at 37 °C overnight. The SA-β-gal signals were analyzed using Image Pro Plus. For each staining assay, the H_2_O_2_-treated HUVECs were used as the positive control.

### Subcellular fractionation

Yeast cells from 50 mL cultures (OD_600_ = 0.7–1.0) were collected, successively washed with ddH_2_O, PBS plus 10 mM β-mercaptoethanol and SB buffer (20 mM Tris pH7.4, 1 M sorbitol) and then suspended in 1 mL SB buffer. 100 µL Zymolase 20 T (10 mg/mL in SB buffer) (mpbiochina) was added and samples were incubated at 30 °C with rotation until > 85% spheroblasts. Spheroblasts were collected by centrifugation at 2000× *g*, washed twice with SB buffer and suspended in 500 µL EBX (20 mM Tris pH7.4, 100 mM NaCl, 0.25% Triton X-100, 15 mM 2-mercaptoethanol, 50 mM sodium butyrate plus protease inhibitors). 0.5% Triton X-100 was then added to lyse the outer cell membrane. An aliquot was taken as the total fraction. The remainder of the lysate was layered over 1 mL NIB (20 mM Tris pH7.4, 100 mM NaCl, 1.2 M sucrose, 15 mM 2-mercaptoethanol, 50 mM sodium butyrate plus protease inhibitors) and centrifuged at 13,000× *g* for 15 min. An aliquot of the upper layer cytoplasmic fraction was taken as the cytoplasm fraction. The glassy white nuclear pellet was suspended in 500 µl EBX and 1% Triton X-100 was added to lyse the nuclear membrane. Samples were kept on ice for 10 min and an aliquot taken as nuclear fraction.

### Co-IP

For Co-IP experiments in yeast, the yeast whole cell extract was prepared by vortexing with glass beads. Pre-cleared cell lysate was incubated with anti-FLAG M2 agarose (Genscript), calmodulin Sepharose beads (GE Healthcare) or indicated antibody conjugated Protein A/G Sepharose beads (GE Healthcare) for 0–4 h at 4 °C. The beads were washed three times with IP washing buffer (40 mM HEPES, pH7.5, 0.1% NP-40, 10% glycerol, 1 mM PMSF, 350 mM NaCl, 2 μg/mL leupeptin, 1 μg/mL pepstatin A) and boiled with 2× SDS-sample buffer for immunoblot analysis.

For Co-IP experiments in mammalian cells, cells were lysed in buffer A (50 mM Tris pH7.4, 150 mM NaCl, 1 mM EDTA, 6 mM sodium deoxycholate, 1% NP-40, 1 mM PMSF, 1:100 proteinase inhibitor cocktail) at 4 °C for 0.5 h. After centrifugation, the supernatant was incubated with indicated antibody conjugated Protein A/G Sepharose beads (GE Healthcare) at 4 °C overnight, then washed with buffer A and boiled with 2× SDS-sample buffer for immunoblots.

### Telomere length quantification

The relative human telomere length was quantified by measuring the amount of telomere repeat amplification product to a single copy gene product (*IFNB1*) using qPCR as described^65,70^. The genome DNA (gDNA) was isolated using the animal genomic DNA kit (Tsingke, Beijing) and quantified by spectrophotometer. 0.5–10 ng gDNA was used in qPCR with SYBR® Green Supermix (Bio-Rad, Catalog #1725121). The primers used were:

*TEL*-F, 5′-CGGTTTGTTTGGGTTTGGGTTTGGGTTTGGGTTTGGGTT-3′;

*TEL*-R, 5′-GGCTTGCCTTACCCTTACCCTTACCCTTACCCTTACCCT-3′;

*IFNB1*-F, 5′-GGTTACCTCCGAAACTGAAGA-3′;

*IFNB1*-R, 5′-CCTTTCATATGCAGTACATTAGCC-3′.

### Statistics and reproducibility

Representative results of at least two biological independent experiments were performed in all of the figure panels. The two-sided Student’s *t*-test was used for comparison between two groups and *P* < 0.05 was considered statistically significant. For boxplots by ggplot2 package, two-sided Wilcoxon test in R (package ggpval) was used for statistical analysis. *P < 0.05, **P < 0.01, ***P < 0.001, n.s., no significance. For all error bars, data represent mean ± SE.

## Supplementary information

Supplemental Information

## Data Availability

All data supporting the findings of this study were included in the manuscript and its supplementary files. The ChIP-seq datasets for Glc7 in YPD and SD-C generated in the current study are available in the GEO repository with the accession number PRJNA866342 and GSE227169. The ChIP-seq datasets for H3pT11/H3 are available in the GEO repository with the accession number PRJNA865151. The GEO accession number for the raw ChIP-seq data for Sen1 in YPD and SD-C is PRJNA866326 and GSE227169. The RNA-seq datasets for *glc7-12* are available in the GEO repository with the accession number PRJNA866675. The RNA-seq datasets for H3T11A and *fbp1Δ* were retrieved from GSE147764 and GSE42526. The mass spectrometry proteomics data have been deposited to the ProteomeXchange Consortium with the dataset identifier PXD036005.
