# Supplementary material for: Glc7/PP1 dephosphorylates histone H3T11 to regulate autophagy and telomere silencing in response to nutrient availability
Source: Cell Discov. 2023 Jul 11;9:71. doi: 10.1038/s41421-023-00551-1 (PMC10336126; doi:10.1038/s41421-023-00551-1)
Supplement: Supplementary file 1 — Supplemental Information [file 41421_2023_551_MOESM1_ESM.pdf]

Supplementary Fig. S1

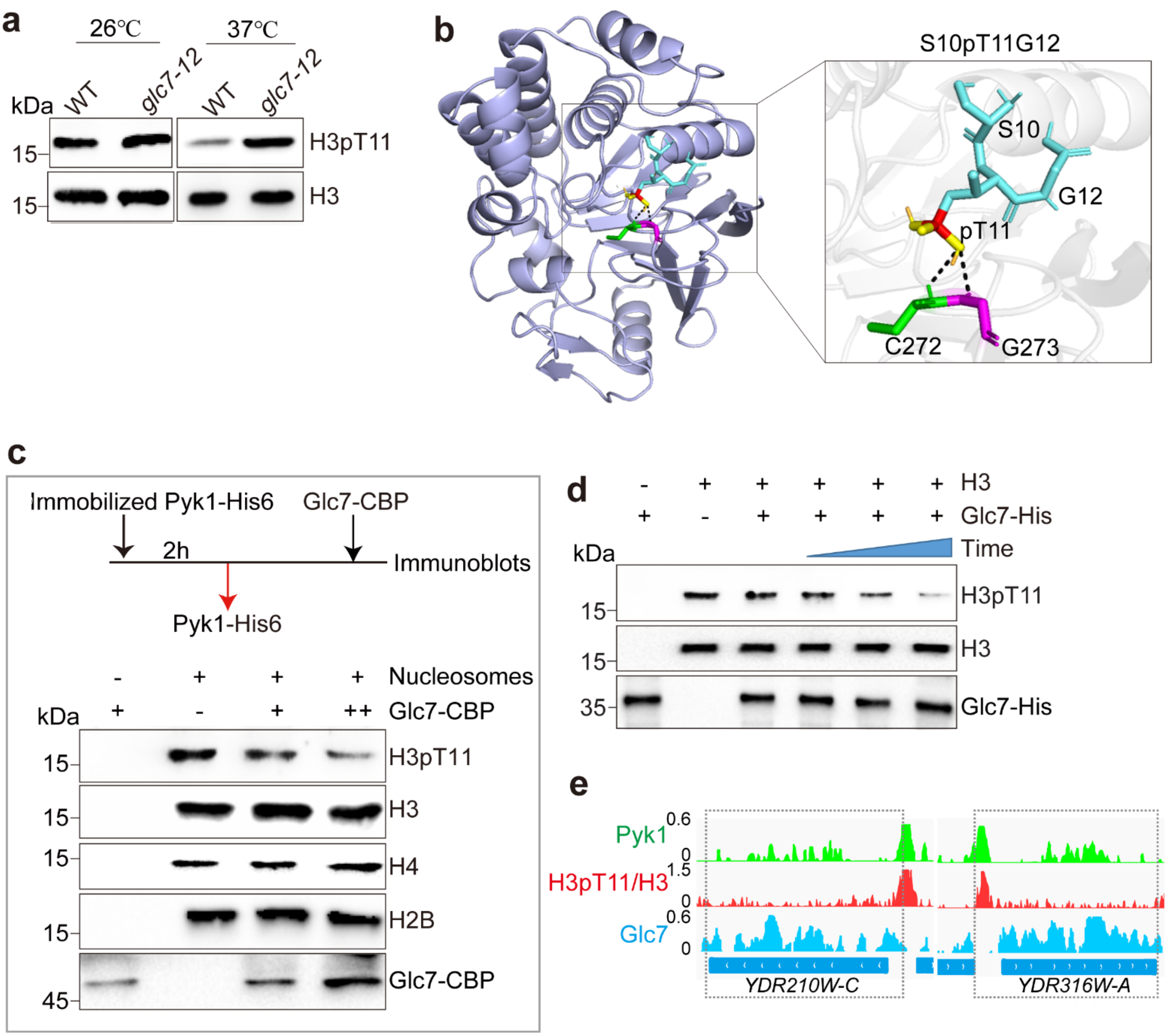

Supplementary Fig. S1 Glc7 dephosphorylates H3T11.

a Immunoblot analysis of H3pT11 in WT and *glc7-12* mutant. Cells were grown at permissive temperature 26 °C until OD_{600} of 0.5 and then treated at non-permissive temperature 37 °C for 2 h. Glc7 was inactivated at 37 °C. **b** Structural view of Glc7 with phosphorylated H3T11 peptide (10-SpTG-12) generated from molecular dynamic simulation**.** **c** *In vitro* dephosphorylation assay showing Glc7-CBP purified from yeast cells dephosphorylates H3T11 within nucleosomes. Nucleosomes were first phosphorylated by Pyk1-His6, which was immobilized on nickel NTA beads. After centrifugation, the nucleosomes-containing supernatant was collected and incubated with Glc7-CBP. **d** *In vitro* dephosphorylation assay showing the purified recombinant Glc7-His dephosphorylates H3T11 within recombinant histone H3. **e** ChIP-seq tracks showing the enrichment of Pyk1, H3pT11/H3 and Glc7 at representative genes.

Supplementary Fig. S2

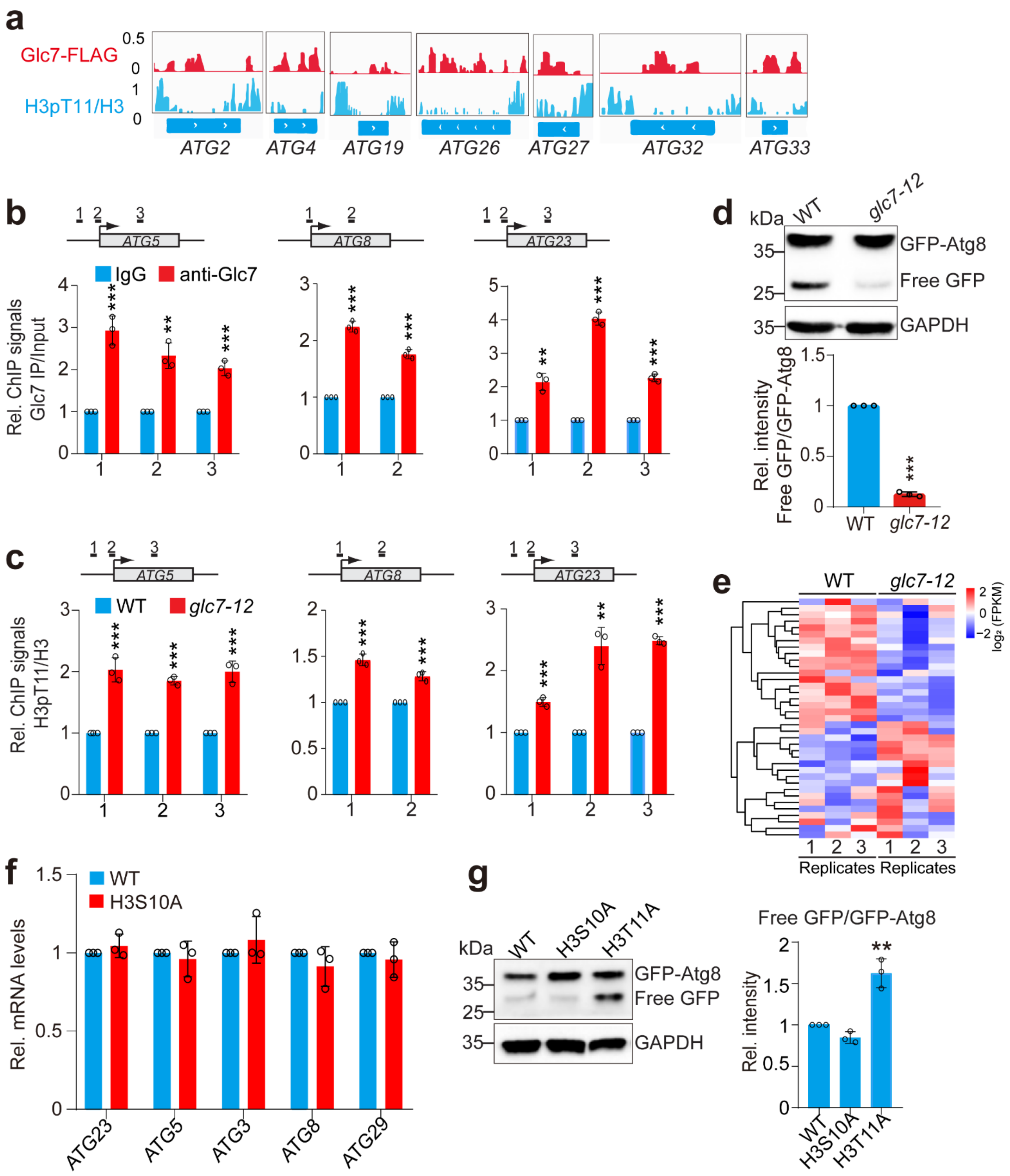

Supplementary Fig. S2 Glc7 dephosphorylates H3T11 to transcriptionally activate autophagy.
a ChIP-seq tracks showing the occupancy of Glc7-FLAG (anti-FLAG) and H3pT11/H3 at representative *ATG* genes. The binding pattern of Glc7-FLAG at *ATG* genes is opposite to that of H3pT11/H3. **b** ChIP-qPCR analysis of Glc7 occupancy at *ATG* genes. Anti-Glc7 antibody was used for ChIP and IgG was served as a negative control. **c** ChIP-qPCR analysis of the occupancy of H3pT11/H3 at *ATG* genes in WT and *glc7-12* mutant. **d** Immunoblot analysis of the autophagy activity in WT and *glc7-12* mutant. **e** Heatmap showing the expression of *ATG* genes in WT and *glc7-12* mutant as determined by RNA-seq analysis. **f** RT-qPCR analysis of the transcription of *ATG* genes in WT and H3S10A mutant. **g** Immunoblot analysis of the autophagy activity in WT, H3S10A and H3T11A mutants.

For Supplementary Fig. S2**c-e**, cells were grown in 26 °C until OD_{600} of 0.5 and then treated at 37 °C for 2 h to inactivate Glc7. For Supplementary Fig. S2**b-d**, **f** and **g**, data represent means ± SE; n=3 independent experiments. Two-sided *t*-tests were used for statistical analysis. *, $P<0.05$; **, $P<0.01$; ***, $P<0.001$.

Supplementary Fig. S3

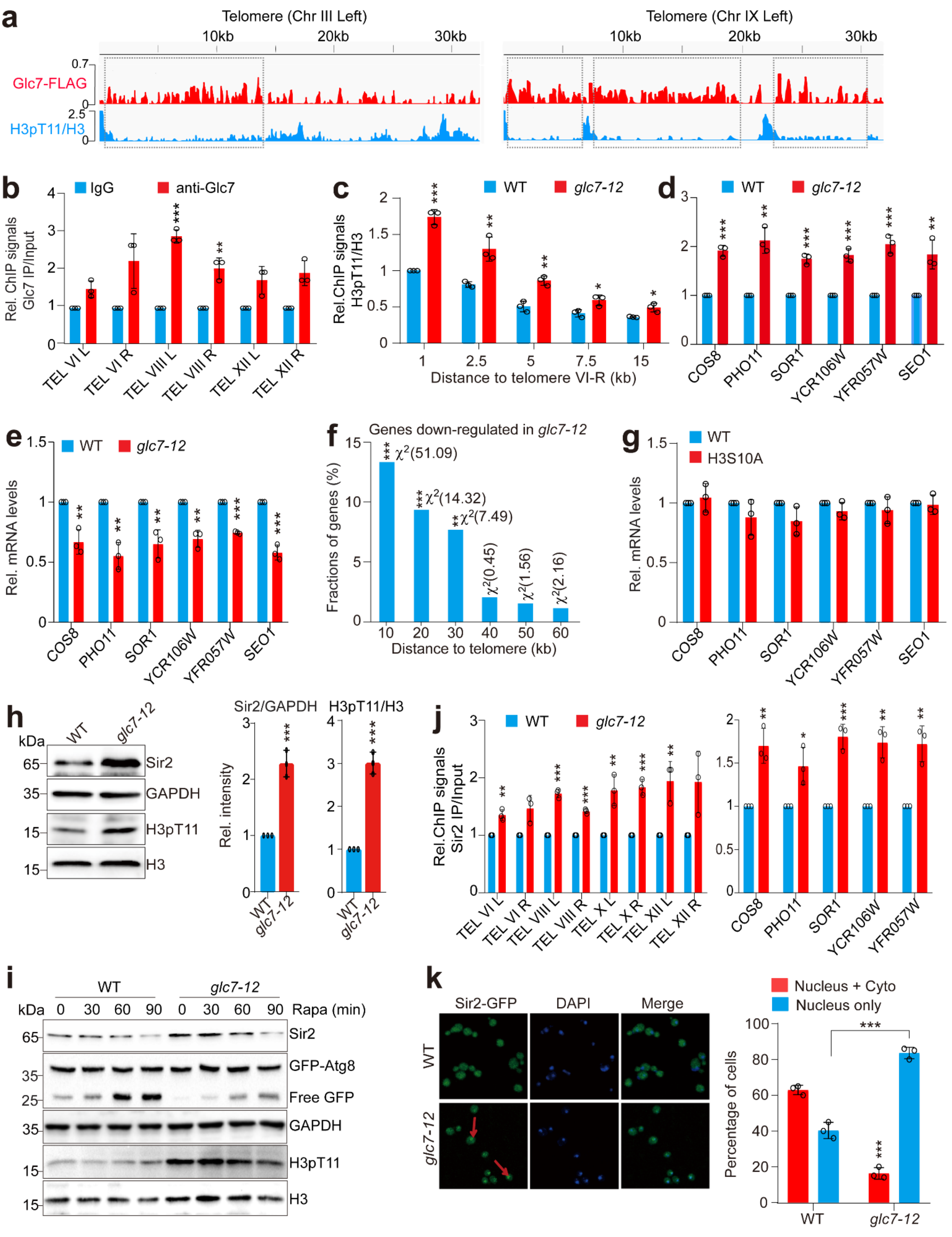

Supplementary Fig. S3 Glc7 dephosphorylates H3T11 to reduce telomere silencing.
a ChIP-seq tracks showing the occupancy of Glc7-FLAG and H3pT11/H3 at representative subtelomere regions. **b** ChIP-qPCR analysis of Glc7 occupancy at the indicated subtelomere regions. **c** ChIP-qPCR analysis of the occupancy of H3pT11/H3 at regions with different distance (1, 2.5, 5, 7.5, and 15 kb) to telomere VI-R in WT and *glc7-12* mutant. **d** ChIP-qPCR analysis of the occupancy of H3pT11/H3 at telomere-proximal genes in WT and *glc7-12* mutant. **e** RT-qPCR analysis the transcription of telomere-proximal genes in WT and *glc7-12* mutant. **f** Histograms showing the proportion of genes down-regulated in *glc7-12* mutant when plotted as a function of their distance to the nearest telomeres. Genes were categorized at 10-kb intervals for up to 60 kb from telomeres. A χ^2 value for each 10-kb interval was calculated by comparing the fraction of genes down-regulated in the interval with the genome-wide average to reflect the telomere-proximal bias of gene down-regulation. **g** RT-qPCR analysis of the transcription of telomere-proximal genes in WT and H3S10A mutant. **h** Immunoblots of Sir2 in WT and *glc7-12* mutant. **i** Immunoblots of Sir2 in WT and *glc7-12* mutant when treated without or with rapamycin for 0-90 min. **j** ChIP-qPCR analysis of Sir2 occupancy at subtelomere regions and telomere-proximal genes in WT and *glc7-12* mutant. **k** Representative fluorescence images showing the distribution of Sir2-GFP (green) in WT and *glc7-12* mutant expressing Sir2-GFP from the native *SIR2* locus. The nucleus DNA was stained with 4',6-diamidino-2-phenylindole (DAPI) as shown in blue. There was less Sir2 diffuse throughout the cell in *glc7-12* mutant compared with WT. More Sir2 was localized in the nucleus in *glc7-12* mutant. Arrows indicate the nucleus-localized Sir2.

For Supplementary Fig. S3**c**-**f**, cells were grown in 26 °C until OD_{600} of 0.5 and then treated at 37 °C for 2 h to inactivate Glc7. For Supplementary Fig. S3**b**-**e**, **g**, **h**, **j** and **k**, data represent means ± SE; n=3 independent experiments. Two-sided *t*-tests were used for statistical analysis. *, *P*<0.05; **, *P*<0.01; ***, *P*<0.001.

Supplementary Fig. S4

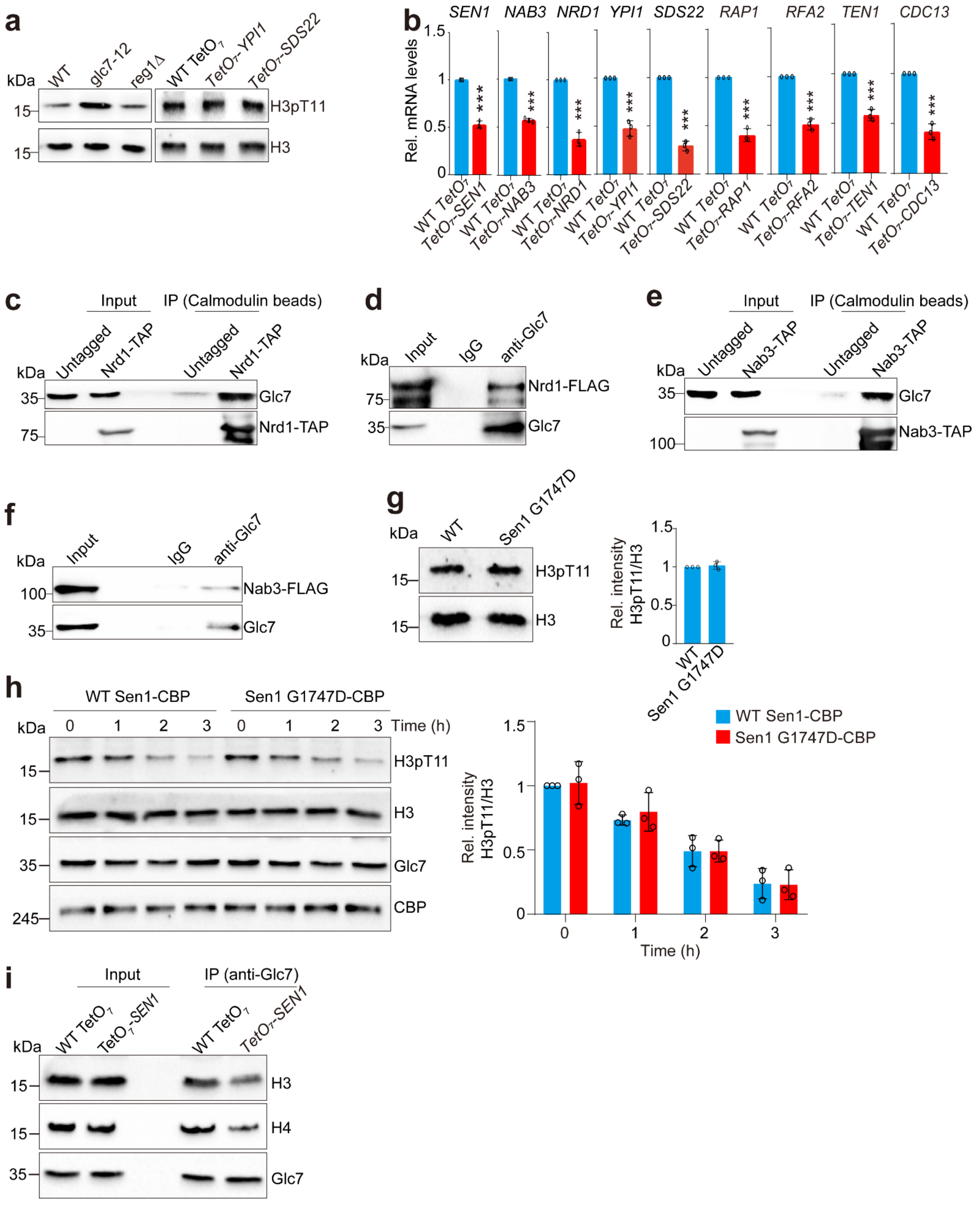

Supplementary Fig. S4 Glc7 interacts with Sen1.

a Immunoblots of H3pT11 in WT, *glc7-12*, *reg1Δ*, WT $TetO_7$, $TetO_7$-*YPI1* and $TetO_7$-*SDS22* mutants. For WT $TetO_7$, $TetO_7$-*YPI1* and $TetO_7$-*SDS22* mutants, cells were grown in YPD medium at 30 ℃ until OD_{600} of 0.5-0.7 and then treated with 40 μg/ml doxycycline for 2 h. **b** RT-qPCR analysis of the knockdown efficiency of indicated genes in WT $TetO_7$, $TetO_7$-*SEN1*, $TetO_7$-*NAB3*, $TetO_7$-*NRD1*, $TetO_7$-*YPI1,* $TetO_7$-*SDS22,* $TetO_7$-*RAP1,* $TetO_7$-*RFA2,* $TetO_7$-*TEN1* and $TetO_7$-*CDC13* mutants. Cells were treated with 40 μg/ml doxycycline for 2 h. **c-d** Co-IP assay and reciprocal IP showing the interaction between Glc7 with Nrd1. Nrd1-TAP was immunoprecipitated by calmodulin beads. **e-f** Co-IP assay and reciprocal IP showing the interaction between Glc7 with Nab3. **g** Immunoblots of H3pT11 in WT and Sen1 G1747D mutant. **h** Sen1 helicase activity is not required for Glc7-mediated H3T11 dephosphorylation. The purified WT Sen1 complex (Sen1-CBP) and Sen1 G1747D complex (Sen1 G1747D-CBP) dephosphorylates H3T11 to the same extent as determined by *in vitro* dephosphorylation assay. **i** Knockdown of Sen1 reduced the interaction between Glc7 and nucleosomes. Glc7 was immunoprecipitated from WT $TetO_7$ and $TetO_7$-*SEN1* mutant with anti-Glc7. The co-immunoprecipitated nucleosomes were probed with anti-H3 and anti-H4 antibodies.

For Supplementary Fig. S4**b**, **g**, **h**, data represent means ± SE; n=3 independent experiments. Two-sided *t*-tests were used for statistical analysis. ***, P<0.001.

Supplementary Fig. S5

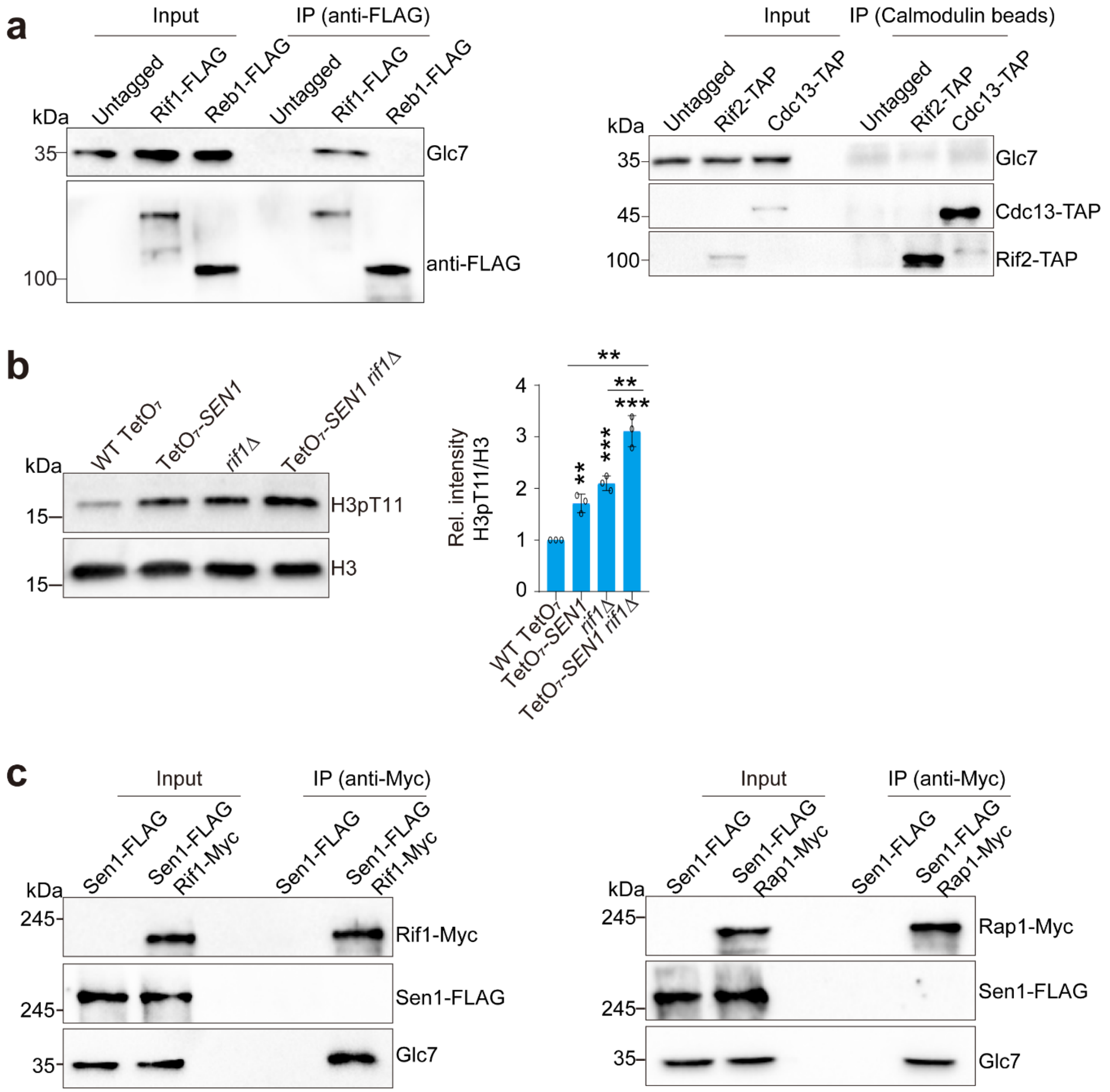

Supplementary Fig. S5 Glc7 interacts with Rif1.
a Co-IP assay and reciprocal IP showing the interaction between Glc7 with Rif1 but not Reb1, Rif2 and Cdc13. **b** Immunoblots of H3pT11 in WT $TetO_7$, $TetO_7$-*SEN1*, *rif1Δ* and $TetO_7$-*SEN1 rif1Δ* mutants. **c** Left panel, Co-IP assay showing that there is no interaction between Sen1 and Rif1. Right panel, Co-IP assay showing that there is no interaction between Sen1 and Rap1.

For Supplementary Fig. S5**b**, data represent means ± SE; n=3 independent experiments. Two-sided *t*-tests were used for statistical analysis. **, $P<0.01$; ***, $P<0.001$.

Supplementary Fig. S6

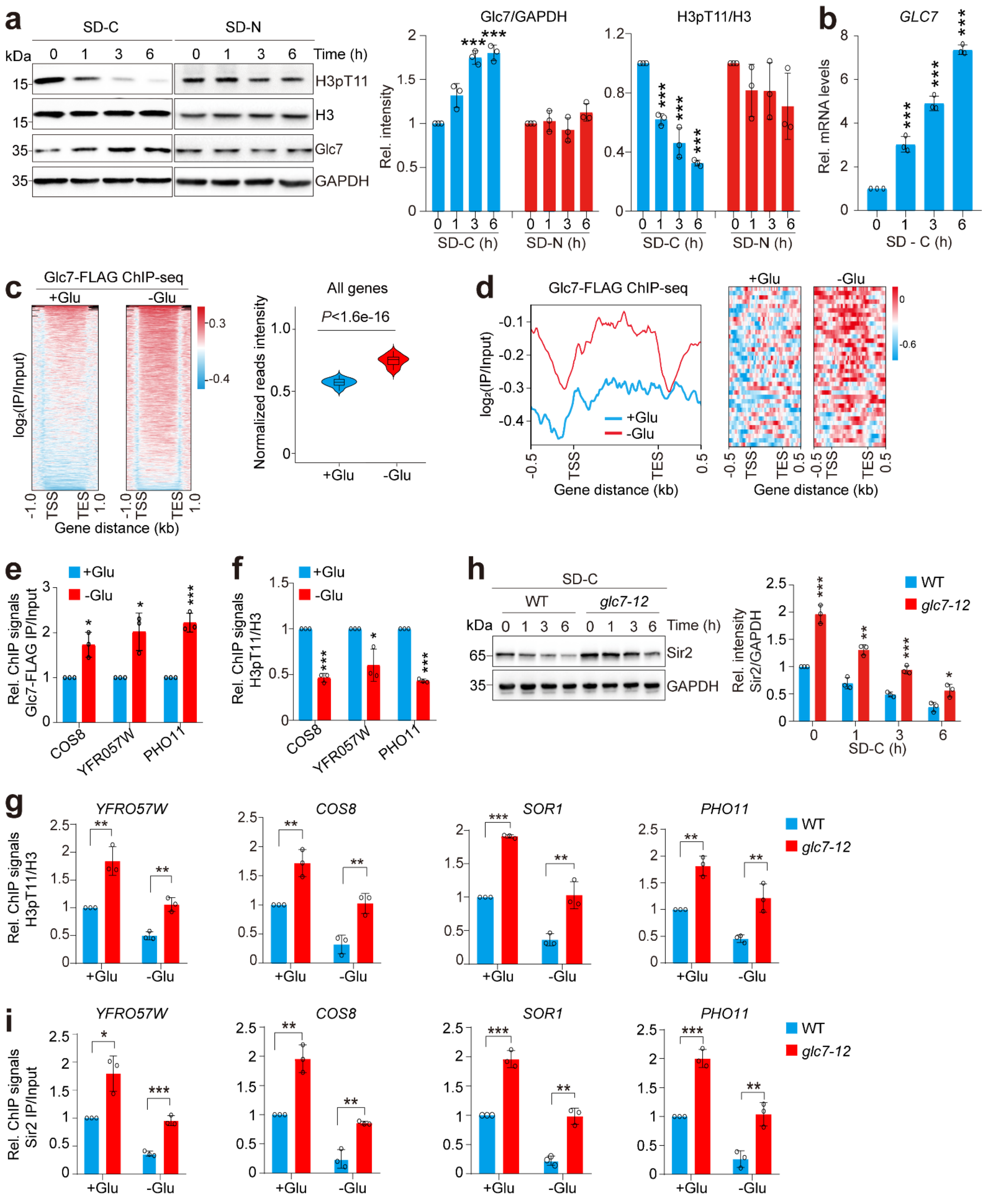

Supplementary Fig. S6 Glc7 dephosphorylates H3T11 to regulate autophagy and telomere silencing under glucose starvation.

a Immunoblots of H3pT11 and Glc7 in cells when grown in SD-C and SD-N medium for 0-6 h, respectively. **b** RT-qPCR analysis of *GLC7* transcription in cells when grown in SD-C medium for 0-6 h. **c** Distribution of Glc7-FLAG occupancy across each gene through 1 kb upstream of the TSS to 1 kb downstream from the TES when cells were grown in SD (+Glu) medium and SD-C (-Glu) medium, respectively. **d** Distribution of Glc7-FLAG occupancy across 39 *ATG* genes through 1 kb upstream of the TSS to 1 kb downstream from the TES when cells were grown in SD (+Glu) medium and SD-C (-Glu) medium, respectively. **e** ChIP-qPCR analysis of Glc7-FLAG occupancy at telomere-proximal genes when cells were grown in SD (+Glu) medium and SD-C (-Glu) medium, respectively. **f** ChIP-qPCR analysis of the occupancy of H3pT11/H3 at telomere-proximal genes when cells were grown in SD (+Glu) medium and SD-C (-Glu) medium, respectively. **g** ChIP-qPCR analysis of the occupancy of H3pT11/H3 at telomere-proximal genes in WT and *glc7-12* mutant when cells were grown in SD (+Glu) medium and SD-C (-Glu) medium at 37 °C for 2 h. **h** Immunoblot analysis of Sir2 in WT and *glc7-12* mutant when grown in SD-C medium at 37 °C for 0-6 h. **i** ChIP-qPCR analysis of Sir2 occupancy at telomere-proximal genes in WT and *glc7-12* mutant when grown in SD (+Glu) medium and SD-C (-Glu) medium at 37 °C for 2 h.

For Supplementary Fig. S6**a**, **b**, **e-i**, data represent means ± SE; n=3 independent experiments. Two-sided *t*-tests were used for statistical analysis. *, $P<0.05$; **, $P<0.01$; ***, $P<0.001$.

Supplementary Fig. S7

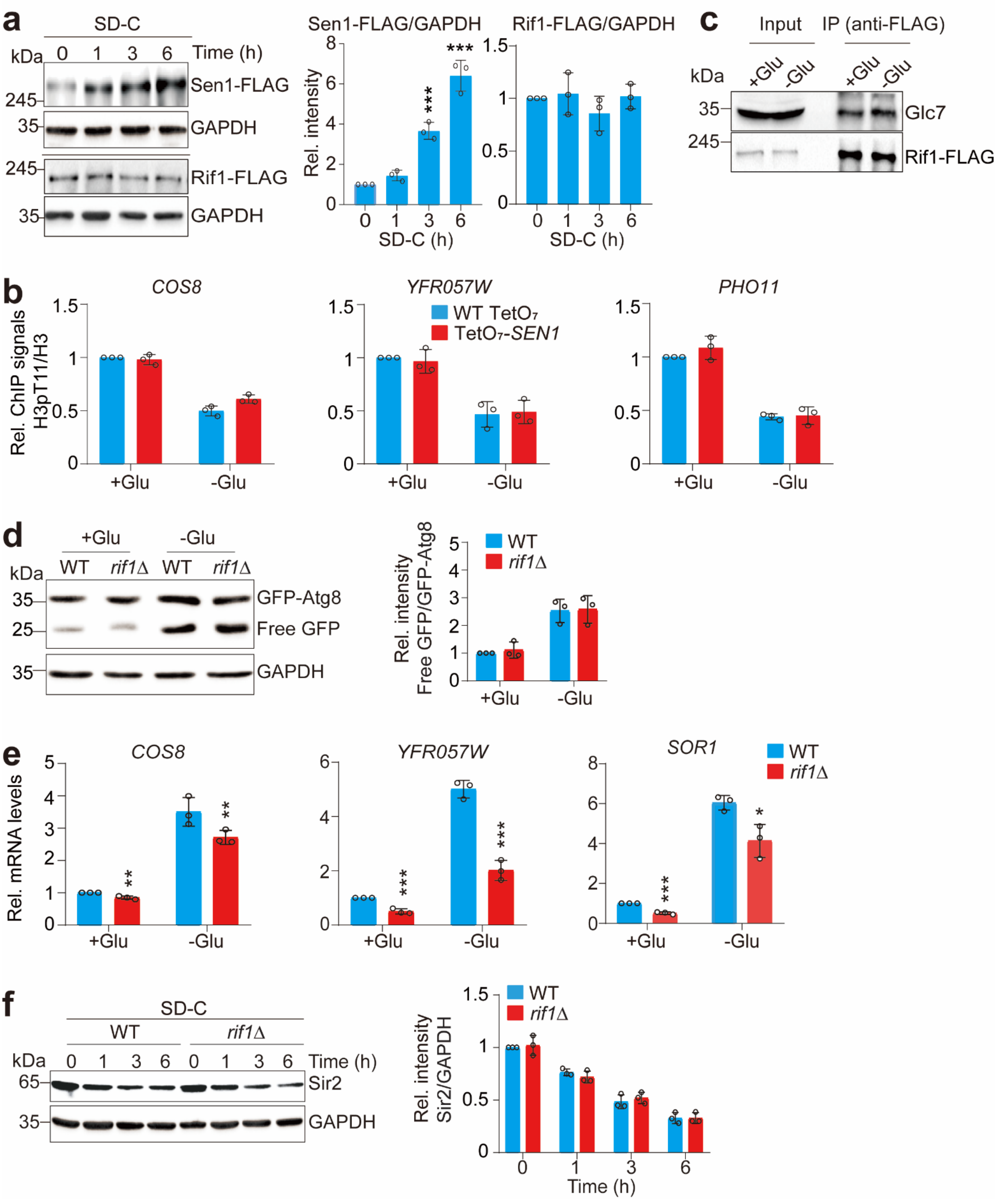

Supplementary Fig. S7 Glc7-Sen1 dephosphorylates H3T11 to regulate autophagy and telomere silencing under glucose starvation.

a Immunoblots of Sen1 and Rif1 when cells were grown in SD-C for 0-6 h. **b** ChIP-qPCR analysis of the occupancy of H3pT11/H3 at telomere-proximal genes in WT $TetO_7$ and $TetO_7$-*SEN1* mutant when grown in SD (+Glu) medium and SD-C (-Glu) medium supplemented with 40 μg/ml doxycycline for 2 h. **c** Co-IP assay showing the interaction between Glc7 and Rif1 was unchanged when cells were grown in SD (+Glu) medium and SD-C (-Glu) medium, respectively. **d** Immunoblot analysis of the autophagy activity in WT and *rif1Δ* mutant when cells were grown in SD (+Glu) medium and SD-C (-Glu) medium, respectively. **e** RT-qPCR analysis of the transcription of telomere-proximal genes in WT and *rif1Δ* mutant when cells were grown in SD (+Glu) medium and SD-C (-Glu) medium, respectively. **f** Immunoblots of Sir2 in WT and *rif1Δ* mutant when cells were grown in SD-C (-Glu) medium for 0-6 h.

For Supplementary Fig. S7**a, b, d-f**, data represent means ± SE; n=3 independent experiments. Two-sided *t*-tests were used for statistical analysis. *, $P<0.05$; **, $P<0.01$; ***, $P<0.001$.

Supplementary Fig. S8

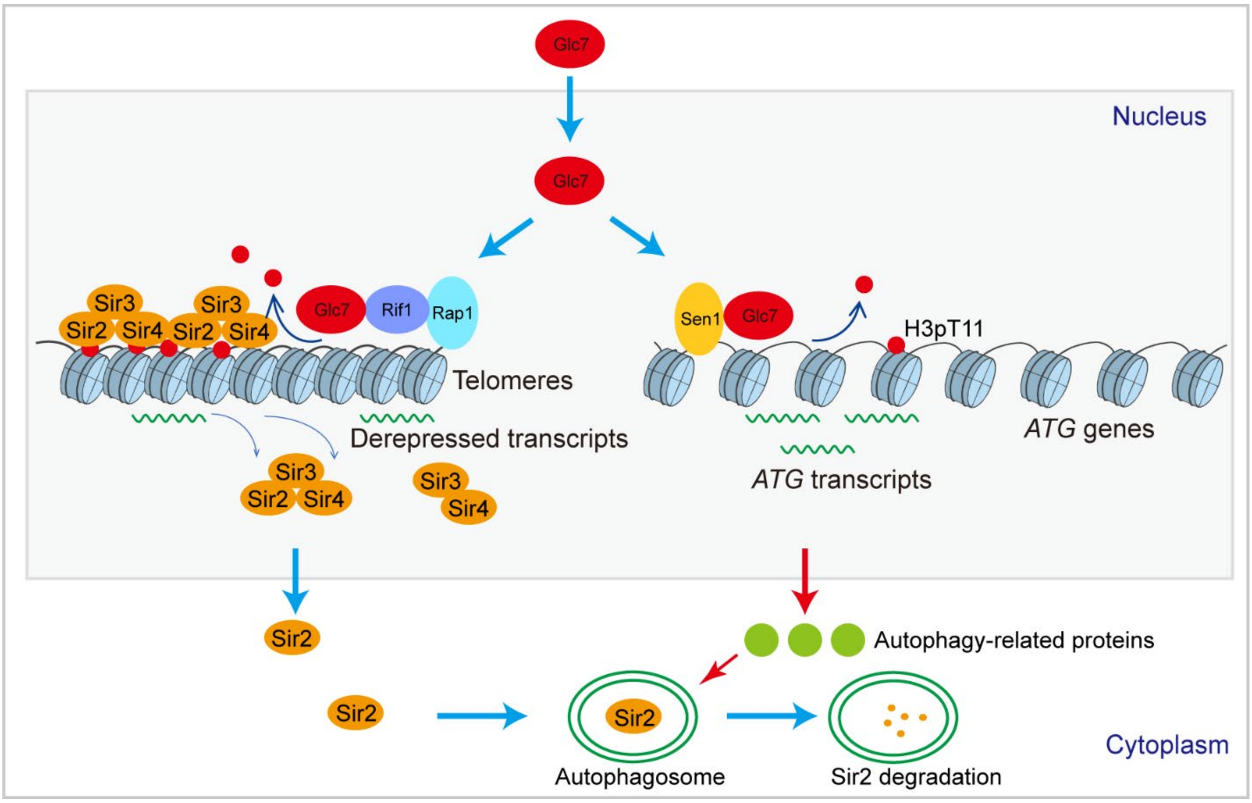

Supplementary Fig. S8 Proposed model for regulation of autophagy and telomere silencing by Glc7/PP1. Under glucose starvation, the expression of Glc7 is increased and more Glc7 translocates into the nucleus, where it is targeted to autophagy-related genes by Sen1 and directed to subtelomere regions by Rap1 and Rif1. Glc7-Sen1 dephosphorylates H3T11 at autophagy-related genes to activate *ATG* gene transcription, which increases autophagy. Glc7-Rif1-Rap1 dephosphorylates subtelomeric H3T11, which dissociates SIR complex from telomere regions, leading to decreased telomere silencing. The dissociated Sir2 is exported into the cytoplasm. Glc7-Sen1 accelerates autophagy and promotes autophagy-mediated degradation of Sir2, which further reduces telomere silencing.

Supplementary Fig. S9

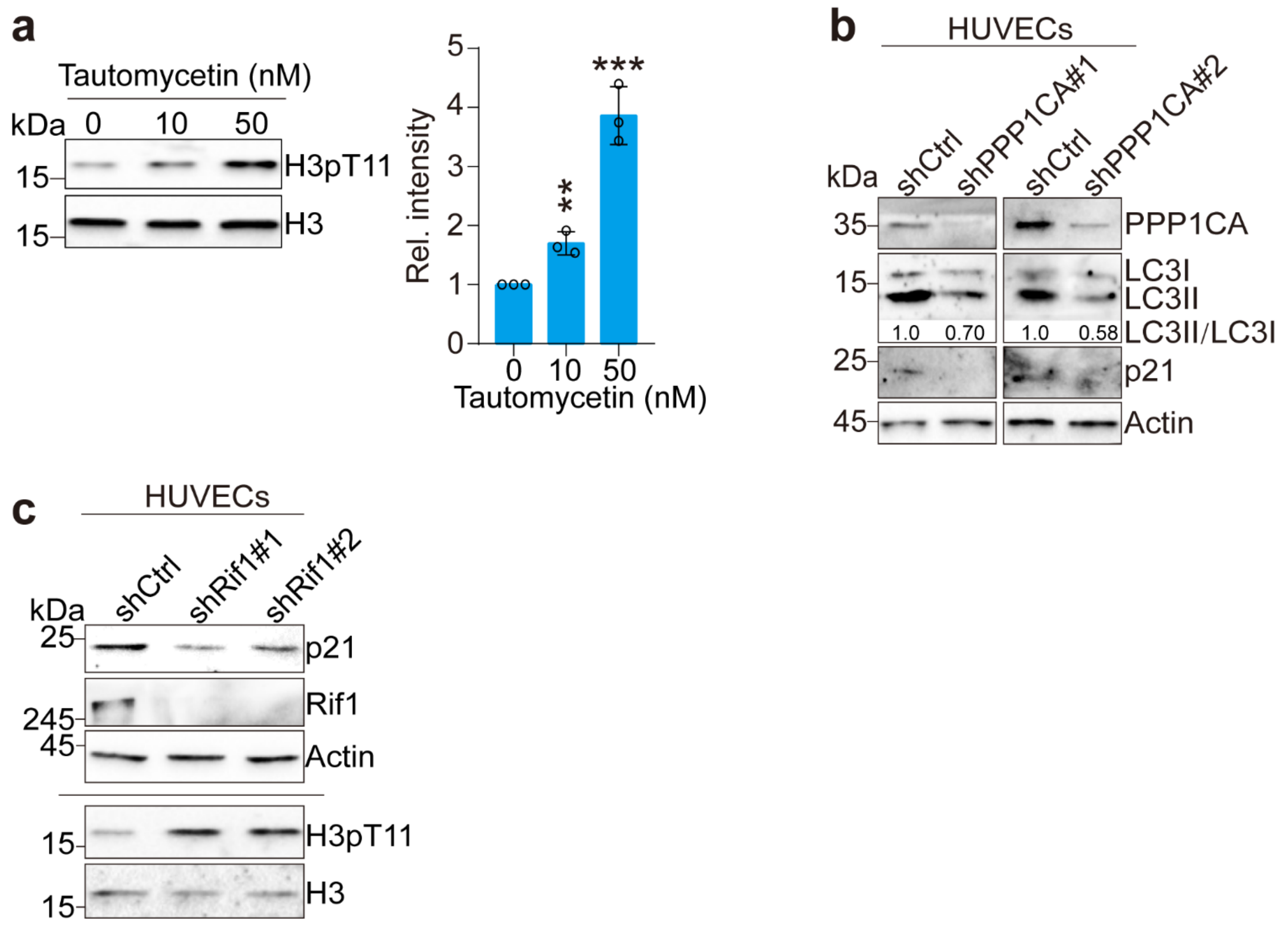

Supplementary Fig. S9 PPP1CA dephosphorylates H3T11 in mammalian cells.
a Immunoblots of H3pT11 in HeLa cells when treated with 0-50 nM tautomycetin for 24 h. **b** Immunoblot analysis of the effect of PPP1CA knockdown on autophagy activity. **c** Immunoblot analysis of p21 and H3pT11 in control (shCtrl) and Rif1-knockdown (shRif1#1, shRif1#2) HUVECs.

For Supplementary Fig. S9**a**, data represent means ± SE; n=3 independent experiments. Two-sided *t*-tests were used for statistical analysis. **, *P*<0.01; ***, *P*<0.001.

Supplementary Fig. S10

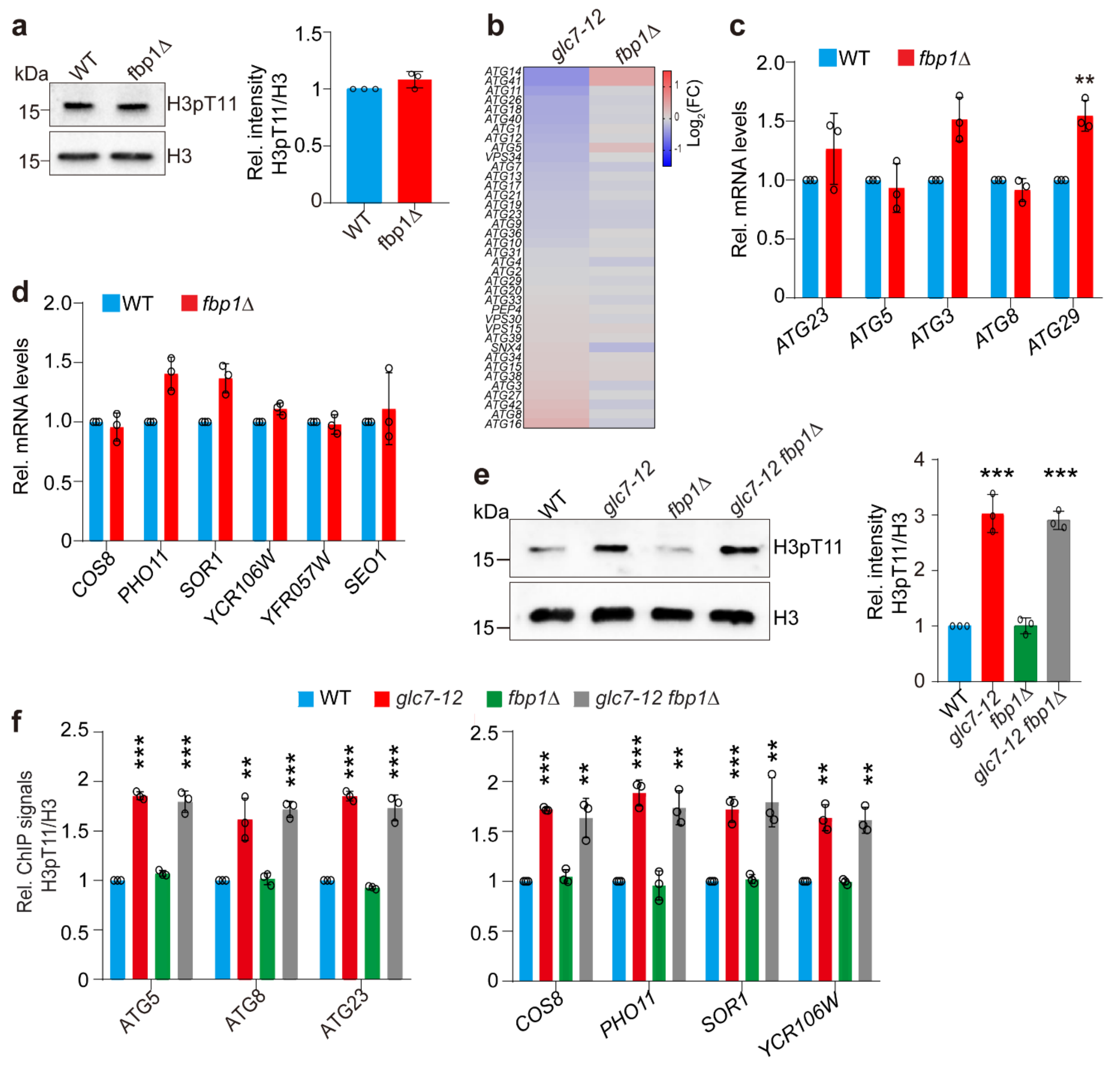

Supplemental Fig. S10 Effect of *FBP1* deletion on H3T11 phosphorylation and autophagy.
a Immunoblot analysis of intracellular H3pT11 levels in WT and *fbp1Δ* mutant. **b** Heatmap showing the transcriptional changes ($\log_2$(mutant/WT)) of *ATG* genes in *fbp1Δ* mutant by RNA-seq. The RNA-seq data for *glc7-12* mutant was used as a control. The RNA-seq data for *fbp1Δ* mutant were retrieved from GSE42526. **c** RT-qPCR analysis of the transcription of indicated autophagy-related genes in WT and *fbp1Δ* mutant. **d** RT-qPCR analysis of the transcription of indicated telomere-proximal genes in WT and *fbp1Δ* mutant. **e** Immunoblot analysis of H3pT11 in WT, *glc7-12*, *fbp1Δ* and *glc7-12 fbp1Δ* mutants. Cells were grown at 37 ºC to inactivate Glc7. **f** Analysis of H3pT11 occupancy at indicated autophagy-related genes and telomere-proximal genes in WT, *glc7-12*, *fbp1Δ* and *glc7-12 fbp1Δ* mutants. Cells were grown at 37 ºC to inactivate Glc7.

For Supplemental Fig. S10**a**, **c-f**, data represent means ± SE; n=3 independent experiments. Two-sided *t*-tests were used for statistical analysis. *, P<0.05; **, P<0.01; ***, P<0.001.

Supplementary Fig. S11

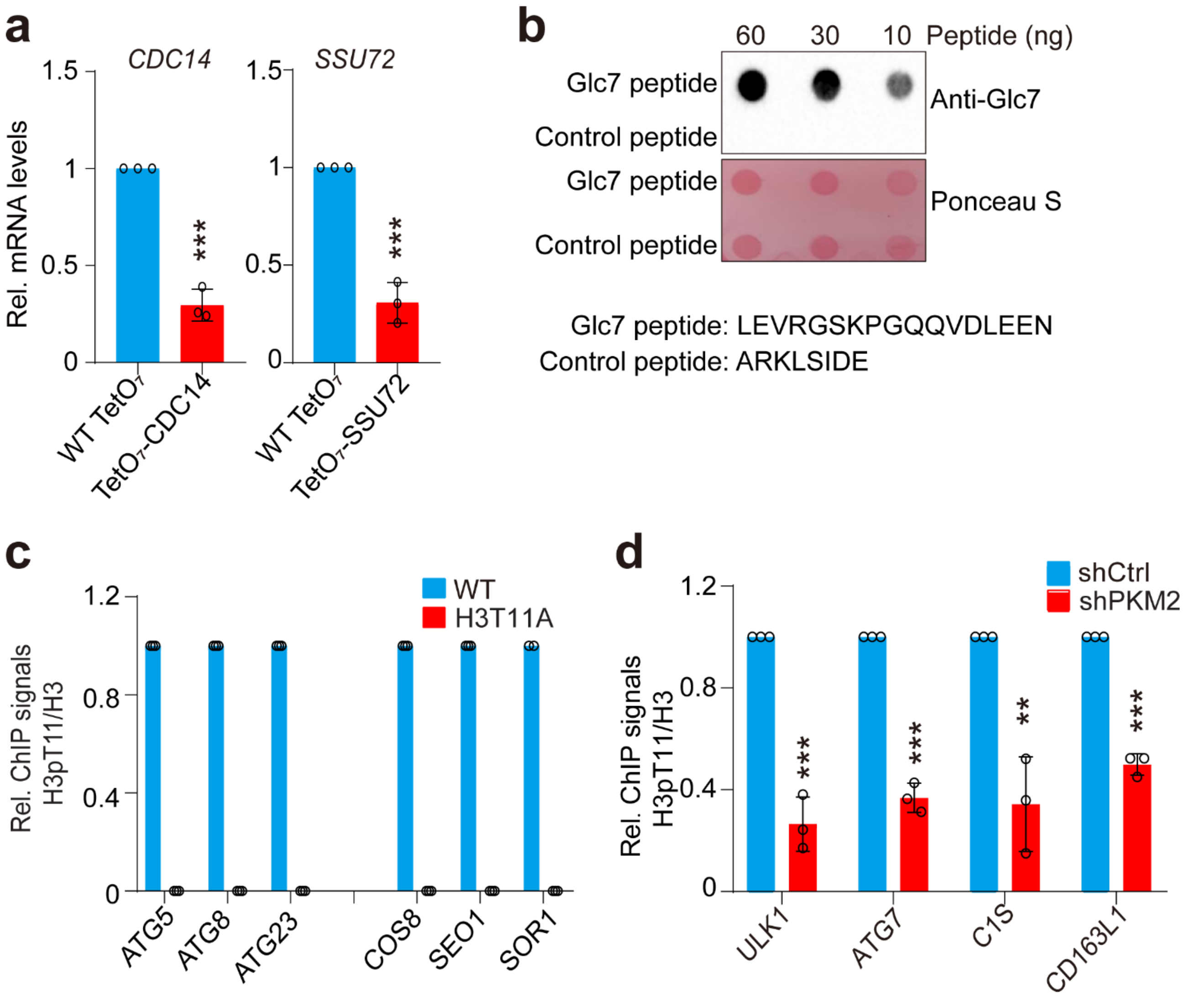

Supplemental Fig. S11 Analysis of knockdown efficiency and antibody specificity. a RT-qPCR analysis of the knockdown efficiency in WT $TetO_7$, $TetO_7$-*CDC14*, and $TetO_7$-*SSU72* mutants. Cells were treated with 40 μg/ml doxycycline for 2 h. **b** Dot blot analysis of the specificity of anti-Glc7 antibody. The serial diluted Glc7 peptide and control peptide were immunoblotted with anti-Glc7 antibody. **c** ChIP-qPCR analysis of H3pT11 occupancy at indicated autophagy-related genes and telomere-proximal genes in WT and H3T11A mutant. **d** ChIP-qPCR analysis of H3pT11 occupancy at indicated autophagy-related genes and telomere-proximal genes in control (shCtrl) and PKM2-knockdown (shPKM2) HUVECs.

For Supplementary Fig. S11**a**, **c-d**, data represent the mean ± SE; n=3 biologically independent experiments. Two-sided *t*-tests were used for statistical analysis. **, $P<0.01$; ***, $P<0.001$.

Supplementary Table S1 List of strains used in this study

Name	Parental Strain	*Genotype*	Source
WT	BY4741	*MATa his3Δ1 leu2Δ0 met15Δ0 ura3Δ0*	Open Biosystems
Sen1-FLAG	BY4741	*MATa his3Δ1 leu2Δ0 met15Δ0 ura3Δ0 Sen1-3xFLAG::KAN*	In this study
Rif1-FLAG	BY4741	*MATa his3Δ1 leu2Δ0 met15Δ0 ura3Δ0 Rif1-3xFLAG::KAN*	In this study
Rap1-FLAG	BY4741	*MATa his3Δ1 leu2Δ0 met15Δ0 ura3Δ0 Rap1-3xFLAG::KAN*	In this study
Sen1-FLAG Rap1-Myc	BY4741	*MATa his3Δ1 leu2Δ0 met15Δ0 ura3Δ0 Sen1-3xFLAG::KAN RAP1-13xMYC::HIS3*	In this study
Sen1-FLAG Rif1-Myc	BY4741	*MATa his3Δ1 leu2Δ0 met15Δ0 ura3Δ0 Sen1-3xFLAG::KAN RIF1-13xMYC::HIS3*	In this study
glc7-ts	BY4741	*MATa his3Δ1 leu2Δ0 met15Δ0 ura3Δ0 glc7-12::KAN hhf1-hht1Δ::HIS3*	Yeast Conditional Temperature Sensitive
BY4741 （*HHF1-HHT1Δ*）	BY4741	*MATa his3Δ1 leu2Δ0 met15Δ0 ura3Δ0 hhf1-hht1::HIS*	In this study
H3T11A (BY4741)	BY4741	*MATa his3Δ1 leu2Δ0 met15Δ0 ura3Δ0 hhf1-hht1::HIS, HHT2(T11A)-HHF2-URA*	In this study
glc7-ts H3T11A	BY4741	*MATa his3Δ1 leu2Δ0 met15Δ0 ura3Δ0 glc7-12::KAN hhf1-hht1Δ::HIS3 HHT2(T11A)-HHF2::URA3*	Yeast Conditional Temperature Sensitive
fbp1Δ	BY4741	*MATa his3Δ1 leu2Δ0 met15Δ0 ura3Δ0 fbp1Δ::KAN*	In this study
glc7-ts fbp1Δ	BY4741	*MATa his3Δ1 leu2Δ0 met15Δ0 ura3Δ0 glc7-12::KAN hhf1-hht1Δ::HIS3 fbp1Δ::LEU*	Yeast Conditional Temperature Sensitive
Glc7-TAP	BY4741	*MATa his3Δ1 leu2Δ0 met15Δ0 ura3Δ0 GLC7-TAP ::HIS3*	In this study
Rif1-TAP	BY4741	*MATa his3Δ1 leu2Δ0 met15Δ0 ura3Δ0 RIF1-TAP ::HIS3*	In this study
Sen1-TAP	BY4741	*MATa his3Δ1 leu2Δ0 met15Δ0 ura3Δ0 SEN1-TAP ::HIS3*	In this study
Nrd1-TAP	BY4741	*MATa his3Δ1 leu2Δ0 met15Δ0 ura3Δ0 NRD1-TAP ::HIS3*	In this study
Nab3-TAP	BY4741	*MATa his3Δ1 leu2Δ0 met15Δ0 ura3Δ0 NAB3-TAP ::HIS3*	In this study
Rif2-TAP	BY4741	*MATa his3Δ1 leu2Δ0 met15Δ0 ura3Δ0 RIF2-TAP ::HIS3*	In this study

CDC13-TAP	BY4741	*MATa his3Δ1 leu2Δ0 met15Δ0 ura3Δ0 CDC13-TAP ::HIS3*	In this study
Sen1 G1747D TAP	BY4741	*MATa his3Δ1 leu2Δ0 met15Δ0 ura3Δ0 SEN1 G1747D-TAP::URA3*	In this study
WT H3	UCC1369	*MATa ade2::hisG his3Δ200 leu2Δ0 lys2Δ0 met15Δ0 trp1Δ63 ura3Δ0 adh4::URA3-TEL-VIIL AdE2-TEL-VR, hhf2-hht2::MET15 hhf1-hht1::LEU2, pDM18-HHT2-HHF2-TRP1*	In this study
H3S10A	UCC1369	*MATa ade2::hisG his3Δ200 leu2Δ0 lys2Δ0 met15Δ0 trp1Δ63 ura3Δ0 adh4::URA3-TEL-VIIL AdE2-TEL-VR, hhf2-hht2::MET15 hhf1-hht1::LEU2, pDM18-HHT2(S10A)-HHF2-TRP1*	In this study
H3T11A	UCC1369	*MATa ade2::hisG his3Δ200 leu2Δ0 lys2Δ0 met15Δ0 trp1Δ63 ura3Δ0 adh4::URA3-TEL-VIIL AdE2-TEL-VR, hhf2-hht2::MET15 hhf1-hht1::LEU2, pDM18-HHT2(T11A)-HHF2-TRP1*	In this study
R1158	R1158	*URA3::CMV-tTA MATa his3-1 leu2-0 met15-0*	Purchase from GE
$TetO_7$-*SEN1*	R1158	*pSEN1::kanR-tetO*$_7$*-TATA URA3::CMV-tTA MATa his3-1 leu2-0 met15-0*	Purchase from GE
$TetO_7$-*NRD1*	R1158	*pNRD1::kanR-tetO*$_7$*-TATA URA3::CMV-tTA MATa his3-1 leu2-0 met15-0*	Purchase from GE
$TetO_7$-*NAB3*	R1158	*pNAB3::kanR-tetO*$_7$*-TATA URA3::CMV-tTA MATa his3-1 leu2-0 met15-0*	Purchase from GE
$TetO_7$-*CDC13*	R1158	*pCDC13::kanR-tetO*$_7$*-TATA URA3::CMV-tTA MATa his3-1 leu2-0 met15-0*	Purchase from GE
$TetO_7$-*TEN1*	R1158	*pTEN1::kanR-tetO*$_7$*-TATA URA3::CMV-tTA MATa his3-1 leu2-0 met15-0*	Purchase from GE
$TetO_7$-*RFA2*	R1158	*pRFA2::kanR-tetO*$_7$*-TATA URA3::CMV-tTA MATa his3-1 leu2-0 met15-0*	Purchase from GE
$TetO_7$-*CDC14*	R1158	*pCDC14::kanR-tetO*$_7$*-TATA URA3::CMV-tTA MATa his3-1 leu2-0 met15-0*	Purchase from GE
$TetO_7$-*SSU77*	R1158	*pSSU77::kanR-tetO*$_7$*-TATA URA3::CMV-tTA MATa his3-1 leu2-0 met15-0*	Purchase from GE
$TetO_7$-*TTS1*	R1158	*pTTS1::kanR-tetO*$_7$*-TATA URA3::CMV-tTA MATa his3-1 leu2-0 met15-0*	Purchase from GE
$TetO_7$-*TAP42*	R1158	*pTAP42::kanR-tetO*$_7$*-TATA URA3::CMV-tTA MATa his3-1 leu2-0 met15-0*	Purchase from GE
$TetO_7$-*RAP1*	R1158	*pRAP1::kanR-tetO*$_7$*-TATA URA3::CMV-tTA MATa his3-1 leu2-0 met15-0*	Purchase from GE
$TetO_7$-*YPI1*	R1158	*pYPI1::kanR-tetO*$_7$*-TATA URA3::CMV-tTA MATa his3-1 leu2-0 met15-0*	Purchase from GE

TetO$_7$-*SDS22*	R1158	*pSDS22::kanR-tetO$_7$-TATA URA3::CMV-tTA MATa his3-1 leu2-0 met15-0*	Purchase from GE
rif1Δ	R1158	*URA3::CMV-tTA MATa his3-1 leu2-0 met15-0 rif1Δ::LEU*	Purchase from GE
TetO$_7$-*SEN1 rif1Δ*	R1158	*pSEN1::kanR-tetO$_7$-TATA URA3::CMV-tTA MATa his3-1 leu2-0 met15-0 rif1Δ::LEU*	Purchase from GE
WT TetO$_7$ ATG8p-GFP-Atg8	R1158	*URA3::CMV-tTA MATa his3-1 leu2-0 met15-0 pFA6a- GFP-ATG8- HIS3*	In this study
TetO$_7$-*SEN1* ATG8p-GFP-Atg8	R1158	*pSEN1::kanR-tetO7-TATA URA3::CMV-tTA MATa his3-1 leu2-0 met15-0 pFA6a- GFP-ATG8- HIS3*	In this study
WT ATG8p-GFP-Atg8	BY4741	*MATa his3Δ1 leu2Δ0 met15Δ0 ura3Δ0 ATG8p-GFP-ATG8-URA3*	In this study
Rif1Δ ATG8p-GFP-Atg8	BY4741	*MATa his3Δ1 leu2Δ0 met15Δ0 ura3Δ0 rif1Δ:: KAN ATG8p-GFP-ATG8-URA3*	In this study
glc7-ts ATG8p-GFP-Atg8	BY4741	*glc7-ts ATG8p-GFP-ATG8-URA3*	Yeast Conditional Temperature Sensitive
tpd3Δ	BY4741	*MATa his3Δ1 leu2Δ0 met15Δ0 ura3Δ0 tpd3Δ::KAN*	In this study
cdc13Δ	BY4741	*MATa his3Δ1 leu2Δ0 met15Δ0 ura3Δ0 cdc13Δ::KAN*	In this study
pph21Δ	BY4741	*MATa his3Δ1 leu2Δ0 met15Δ0 ura3Δ0 pph21Δ::KAN*	In this study
pph22Δ	BY4741	*MATa his3Δ1 leu2Δ0 met15Δ0 ura3Δ0 pph22Δ::KAN*	In this study
sit4Δ	BY4741	*MATa his3Δ1 leu2Δ0 met15Δ0 ura3Δ0 sit4Δ::KAN*	In this study
pph1Δ	BY4741	*MATa his3Δ1 leu2Δ0 met15Δ0 ura3Δ0 pph1Δ::KAN*	In this study
ppg1Δ	BY4741	*MATa his3Δ1 leu2Δ0 met15Δ0 ura3Δ0 ppg1Δ::KAN*	In this study
ppz1Δ	BY4741	*MATa his3Δ1 leu2Δ0 met15Δ0 ura3Δ0 ppz1Δ::KAN*	In this study
ppz2Δ	BY4741	*MATa his3Δ1 leu2Δ0 met15Δ0 ura3Δ0 ppz2Δ::KAN*	In this study
ppq1Δ	BY4741	*MATa his3Δ1 leu2Δ0 met15Δ0 ura3Δ0 ppq1Δ::KAN*	In this study
cna1Δ	BY4741	*MATa his3Δ1 leu2Δ0 met15Δ0 ura3Δ0 cna1Δ::KAN*	In this study

cmp2Δ	BY4741	*MATa his3Δ1 leu2Δ0 met15Δ0 ura3Δ0 cmp2Δ::KAN*	In this study
msg5Δ	BY4741	*MATa his3Δ1 leu2Δ0 met15Δ0 ura3Δ0 msg5Δ::KAN*	In this study
sdp1Δ	BY4741	*MATa his3Δ1 leu2Δ0 met15Δ0 ura3Δ0 sdp1Δ::KAN*	In this study
yvh1Δ	BY4741	*MATa his3Δ1 leu2Δ0 met15Δ0 ura3Δ0 yyh1Δ::KAN*	In this study
pps1Δ	BY4741	*MATa his3Δ1 leu2Δ0 met15Δ0 ura3Δ0 pps1Δ::KAN*	In this study
tep1Δ	BY4741	*MATa his3Δ1 leu2Δ0 met15Δ0 ura3Δ0 tep1Δ::KAN*	In this study
mih1Δ	BY4741	*MATa his3Δ1 leu2Δ0 met15Δ0 ura3Δ0 mih1Δ::KAN*	In this study
ych1Δ	BY4741	*MATa his3Δ1 leu2Δ0 met15Δ0 ura3Δ0 ych1Δ::KAN*	In this study
ltp1Δ	BY4741	*MATa his3Δ1 leu2Δ0 met15Δ0 ura3Δ0 ltp1Δ::KAN*	In this study
oca1Δ	BY4741	*MATa his3Δ1 leu2Δ0 met15Δ0 ura3Δ0 oca1Δ::KAN*	In this study
oca2Δ	BY4741	*MATa his3Δ1 leu2Δ0 met15Δ0 ura3Δ0 oca2Δ::KAN*	In this study
siw14Δ	BY4741	*MATa his3Δ1 leu2Δ0 met15Δ0 ura3Δ0 siw4Δ::KAN*	In this study
ptp1Δ	BY4741	*MATa his3Δ1 leu2Δ0 met15Δ0 ura3Δ0 ptp1Δ::KAN*	In this study
ptp2Δ	BY4741	*MATa his3Δ1 leu2Δ0 met15Δ0 ura3Δ0 ptp2Δ::KAN*	In this study
ptp3Δ	BY4741	*MATa his3Δ1 leu2Δ0 met15Δ0 ura3Δ0 ptp3Δ::KAN*	In this study
ymr1Δ	BY4741	*MATa his3Δ1 leu2Δ0 met15Δ0 ura3Δ0 ymr1Δ::KAN*	In this study
rtr1Δ	BY4741	*MATa his3Δ1 leu2Δ0 met15Δ0 ura3Δ0 rtr1Δ::KAN*	In this study
rtr2Δ	BY4741	*MATa his3Δ1 leu2Δ0 met15Δ0 ura3Δ0 rtr2Δ::KAN*	In this study
ptc1Δ	BY4741	*MATa his3Δ1 leu2Δ0 met15Δ0 ura3Δ0 ptc1Δ::KAN*	In this study
ptc2Δ	BY4741	*MATa his3Δ1 leu2Δ0 met15Δ0 ura3Δ0 ptc2Δ::KAN*	In this study
ptc3Δ	BY4741	*MATa his3Δ1 leu2Δ0 met15Δ0 ura3Δ0 ptc3Δ::KAN*	In this study
ptc4Δ	BY4741	*MATa his3Δ1 leu2Δ0 met15Δ0 ura3Δ0 ptc4Δ::KAN*	In this study

ptc5Δ	BY4741	*MATa his3Δ1 leu2Δ0 met15Δ0 ura3Δ0 ptc5Δ::KAN*	In this study
ptc6Δ	BY4741	*MATa his3Δ1 leu2Δ0 met15Δ0 ura3Δ0 ptc6Δ::KAN*	In this study
ptc7Δ	BY4741	*MATa his3Δ1 leu2Δ0 met15Δ0 ura3Δ0 ptc7Δ::KAN*	In this study
nem1Δ	BY4741	*MATa his3Δ1 leu2Δ0 met15Δ0 ura3Δ0 nem1Δ::KAN*	In this study
psr1Δ	BY4741	*MATa his3Δ1 leu2Δ0 met15Δ0 ura3Δ0 psr1Δ::KAN*	In this study
psr2Δ	BY4741	*MATa his3Δ1 leu2Δ0 met15Δ0 ura3Δ0 psr2Δ::KAN*	In this study
reg1Δ	BY4741	*MATa his3Δ1 leu2Δ0 met15Δ0 ura3Δ0 reg1Δ::KAN*	In this study
rif1Δ	BY4741	*MATa his3Δ1 leu2Δ0 met15Δ0 ura3Δ0 rif1Δ::KAN*	In this study
rif2Δ	BY4741	*MATa his3Δ1 leu2Δ0 met15Δ0 ura3Δ0 rif2Δ::KAN*	In this study
ku70Δ	BY4741	*MATa his3Δ1 leu2Δ0 met15Δ0 ura3Δ0 ku70Δ::KAN*	In this study
ku80Δ	BY4741	*MATa his3Δ1 leu2Δ0 met15Δ0 ura3Δ0 ku80Δ::KAN*	In this study
est1Δ	BY4741	*MATa his3Δ1 leu2Δ0 met15Δ0 ura3Δ0 est1Δ::KAN*	In this study
est2Δ	BY4741	*MATa his3Δ1 leu2Δ0 met15Δ0 ura3Δ0 est2Δ::KAN*	In this study
est3Δ	BY4741	*MATa his3Δ1 leu2Δ0 met15Δ0 ura3Δ0 est3Δ::KAN*	In this study

Supplementary Table S2 List of oligonucleotides used in this study

Gene name	Sequence
**qRT-PCR (Yeast)**	
ATG23	CTCCATAGCGAAAGTACCACTG<br>CGACCCTTTATGGCTTTTGTG
ATG5	ACCAGGTAAAGGATGTTCTCAC<br>TGCGATGGGAATGATAGTTGG
ATG38	CAAATGCAAAAGCGAAGTATCAAG<br>ATGGCTTGCGTAACGTCA
ATG29	CTATTAAATGTATCCGCAAGCCC<br>CGCCTCATTTGTTACTTCTGTC
ATG9	AGCAACTTCCCTTTACCAGAC<br>GTCAGACTCAGGAACACGTAAG
ATG11	AACTCCCCTAATTCCAACGAC<br>AACGCGAAAGATCTACGTCTG
ATG8	ACCTTACCGTAGGGCAATTTG<br>CCCGTCCTTATCCTTGTGTTC
ATG3	TTGCTAGATAAGGTTCGTGTGG<br>CAAGTATTGGTCTACCCGTAACG
ATG14	CAAGATGAAGTGTAGGTCCGTC<br>CATGAGGTCCTGTGACTGTTG
ATG10	TGCAATCGTTGTATGACTCCC<br>TTTCTGCAGTTGTCGAGTAGG
ATG17	AGGAGAAAGGATGTGGCAAAC<br>TTTACCGGGCCAGATTGTC
ATG7	TCCCTGTTTTCAAAGACCCTC<br>GAGAACAGCCCTTTTAAACTCG
ATG12	GGAACGGCAATGGAAAGATC<br>TCAACTTGCTGGTCGACAG
ACTIN	TCGAACAAGAAATGCAAACCG<br>GGCAGATTCCAAACCCAAAAC
PMA1	AAGATCACCCAATCCCAGAAG<br>ACCCAAGATTTCCCAGTGAC
SOR1	GTAGTTCTAGAGAAAGTCGGCG<br>GGCGCCTTCAATATGTACTTACC
YCR106W	CGCCTTAGGGTTATTATACAATGC<br>CGCTCTCAAAGAGTGAAATGTCC
PHO11	GACAAAATCGGAACTCAAACGG<br>TCTTTCACCGTGTCTACCAAC

COS8	CCGTTCTACCTCAAGATGTTTTCCG<br>CCAGGAACAGGACAAGAAGTGAAAC
YFR057W	TGATATTTGGACCTACTAGTGTCTATAG<br>GCTTGGCGGTGTCTTTAATG
SEN1	ATCAATACTCAACGGCTCCTG<br>GAAGATTGTGGTTGCGGATG
RAP1	GAAAACACTGGTGCTACTGC<br>AGTATCAGCAATGGAAGGCG
NRD1	TTGACTTCCACAGATCCAGC<br>TCGGTAATTGTATGGGCTTGG
NAB3	CAAGGGTATGGTCGTTATCAGG<br>GTGGAAGGTTTTGAATGGCAG
SDS22	ATCCTTTGAGAGCTTGGGTG<br>CTGTAGGGATGGAGGTAAGTTC
YPI1	GTGGAAATCAAATGGCTATGGG<br>AGGATCTTGAGTTGCTCGAAG
TAF2	GAAAGTGACGAAGAGGAAGAGG<br>AATCACATCCCTCAAACTCGG
TOA2	CTGGACACATATGGATTTTGCG<br>ACAATCCTCAACTTATCCACCG
SUA7	GGAGAGCATAGATAAAAGAGCAGG<br>AGACCACATAGAGCACATACAAC
JEM1	TCCAACGATCAACATCTAAGAGC<br>TCCCACATCCCAAAATCGAC
SLG1	CAGCTCAGATTCAAGTACGGAG<br>CTGTAGAGGAAGACGCCATTG
ENO2	CTCCAAACATTCAAACCGCTG<br>GTCGTACTTACCGTCCTTGAAG
DIS3	CCCCTAAGTTTGACTCATCGTG<br>TTCCTCATTACTTGCCCGAC
RRP6	CCGCCAGAATGCAAAACTATTAG<br>ATTCCGTCTGCTTTGGTCTC
AIR1	TGCTAACCAAGGTGACTTCG<br>GCACAGTCATCGCCAAAATG
ACC1	CTGAAGATGGGTTAGGTGTCG<br>GACCCAAACGAACCAAATAAGC
RPB1-1	TTCTTACTCTCCCACCTCTCC<br>TTTTGTTCGTCTTGCTTTGGAG
KIN28	GCTAACGATGAACCCACAAAAG<br>ATTGAAGACGGGTCACTTGG

qRT-PCR (Mammalian cells)	
BECN1	CTACCGGGAAGTCGCTGA<br>GTCACCCAAGTCCGGTCTAC
ATP6V0D2	TTTTGGCTAATCACACAAATCC<br>AGGTGAGAAATGTGCTGAGG
FOXO3	CCTTGTACAGGGTGGTGAGC<br>AAACGTCCCATAAACCATCG
ATG13	GCGGAGTCTTAGGAGCAAAA<br>GGTTCCGGGCTCTCATTAC
ULK1	ACACCATCAGGCTCTTCCTG<br>GATCTTGACGCGGATGCT
ATG3	ACGGCAAGAGAGTGAGAAGG<br>GAAGGGAGACCTGAGGTGAG
ATG4B	ACTGCGTTTCCTGCAGATTC<br>ACGTAGGCCTCGTTGATGTC
ATG7	GGGGGATCCTGGACTCTCTA<br>GAGCTTCATCCAGCCGATAC
ATG12	GGCGTTTTGGTTTCACATCT<br>TGTCCTATGTGCTTGCTCTCC
RIF1	TGTTGGAGACTTTGGAAGACC<br>ACTTTGTACAGCCGAGGAAG
SETX	CTTCATCCTCGGACATTTGAG<br>TTAATAATGGCACCACGCTTC
PPP1CA	GCCTATAAGATCAAGTACCCCG<br>AGTCAGTGAAGGTTTTCCACAG
ACTIN	GCCGACAGGATGCAGAAGGAGATCA<br>AAGCATTTGCGGTGGACGATGGA
P21	GACACCACTGGAGGGTGACT<br>CAGGTCCACATGGTCTTCCT
P16	CCAACGCACCGAATAGTTACG<br>GCGCTGCCCATCATCATG
IL1A	AGTGCTGCTGAAGGAGATGCCTGA<br>CCCCTGCCAAGCACACCCAGTA
IL1B	TGCACGCTCCGGGACTCACA<br>CATGGAGAACACCACTTGTTGCTCC
IL-6	CCGGGAACGAAAGAGAAGCT<br>GCGCTTGTGGAGAAGGAGTT
IL-8	ACTTCTCCACAACCCTCTG<br>TACTCCAAACCTTTCCACC

CCND2	CCTCCAAACTCAAAGAGACCAG<br>TTCCACTTCAACTTCCCCAG
C1S	TTTGTAGATGTCCCTTGTAGCC<br>AATCTCCCCAATCAGTGCAG
CDL1163L1	TGGACACAAGGAAGATGCTG<br>GGAGAAGGAGCCCAAAGATAC
**ChIP-qPCR (Yeast)**	
ATG5-1	AACTGTGGCAAGAACATCGC<br>AGGCGTTATGTTTCTCATCGC
ATG5-2	AGCATGCTCAGAAGTGCGAA<br>ACATCATAGGTTTCCTATCTCCGT
ATG5-3	ACCAGGTAAAGGATGTTCTCAC<br>TGCGATGGGAATGATAGTTGG
ATG8-1	ACCCGTGAAATCATAGCACAT<br>ACCAAATATTTTTGCCGCCG
ATG8-2	TAAAGGTTGAGGAGGGGAT<br>ATTATGATTTTCTCAACTTTAC
ATG23-1	ATAGAACACAGAGAACGTCTG<br>CCATTCCTTAGCCGGTTCTCC
ATG23-2	AGTTCAAGTGATTATTTTCGTTTTCTTGT<br>CTTCTTCACTTTATTTGTTACCTTATAGAA
ATG23-3	CTCCATAGCGAAAGTACCACTG<br>CGACCCTTTATGGCTTTTGTG
ChrVI L	CCACACCCACACCCTAATAC<br>GGGTAATGGAGGGTAAGTTGAG
ChrVI R	AGGATTATAGGTAAATGGCAAGGG<br>GACCCAGTCCTCATTTCCATC
ChrVIII L	TTACCCCATCTAAAGTGCCG<br>ACAACCACACCTCCGAAATC
ChrVIII R	CACACCCACACTTTTCACATC<br>GCTATGTAGAAGTGCTGTAGGG
ChrX R	CAGCATTCTCTCCACAGCTAGT<br>CGGATTATGCAAGGGCTGTCAA
ChrX L	TTACCCCATCTAAAGTGCCG<br>ACAACCACACCTCCGAAATC
ChrXIIL	GGTACGGTGGTACTCAGATCAT<br>GAATCTCACACTGACGCATGAT
ChrXII R	ATCTACCTCTACTCTCGCTGTC<br>CGTACATGAGGGCTATTTAGGG
**ChIP-qPCR (Mammalian cells)**	
IFNB1	GGTTACCTCCGAAACTGAAGA<br>CCTTTCATATGCAGTACATTAGCC
TEL	CGGTTTGTTTGGGTTTGGGTTTGGGTTTGGGTTTGGGTT

	GGCTTGCCTTACCCTTACCCTTACCCTTACCCTTACCCT
ULK1-Pro	TGCCCTGTTCCATATTTTGC<br>ACCCAAACCAACGACATAGC
ATG7-Pro	GCAGTCATCGCTCTTGTTGTTATG<br>GGGTGGCAGGTGTGGAGAG
ATG12-Pro	GTGGGCTCTTGGGGACTG<br>GGTGGGCTGCATGCTTTC
CCND2-Pro	GGGTGACGGGAGGAAGGAGGTGA<br>GGGAGGGGGCGAGTGAGGGATTA
C1S-Pro	GAGTCTGGGTTGGATCTGAAAG<br>CATAACATCCCTCCACATCCC
CDL1163L1-Pro	TCCTGGCAAACACTCTGATG<br>CTCCCCGATTACAGTGACAAG

Supplementary Table S3 List of siRNA and shRNA used in this study

Gene name	Sequence
SETX#1	5'- AGCAAGAGAUGAAUUGCCA-3'
SETX#2	5'- GCTCAACTCTCCAAATAGA-3'
PPP1CA#1	5'- CCGGGCAAGAGACGCTACAACATCACTCGA GTGATGTTGTAGCGTCTCTTGCTTTTTG -3'
	5'-AATTCAAAAAGCAAGAGACGCTACAACATC ACTCGAGTGATGTTGTAGCGTCTCTTGC -3'
PPP1CA#2	5'- CCGGGAAGACGGCTACGAGTTCTCTCGAGA GAACTCGTAGCCGTCTTCTTTTTG -3'
	5'- CCGGGAAGACGGCTACGAGTTCTCTCGAGA GAACTCGTAGCCGTCTTC TTTTTG -3'
Rif1#1	5'- CCGGAATGAGACTTACGTGTTAAAACTCGA GTTTTAACACGTAAGTCTCATTTTTTTG -3'
	5'- AATTCAAAAAAAATGAGACTTACGTGTTAA AACTCGAGTTTTAACACGTAAGTCTCATT -3'
Rif1#2	5'- CCGGAAGAGAAACCAGGTTCTGAAGCTCG AGCTTCAGAACCTGGTTTCTCTTTTTTTG -3'
	5'- AATTCAAAAAAAAGAGAAACCAGGTTCTG AAGCTCGAGCTTCAGAACCTGGTTTCTCTT -3'
SETX	5'- CCGGGCTCAACTCTCCAAATAGACTCGAGT CTATTTGGAGAGTTGAGCTTTTTG -3'
	5'- AATTCAAAAAGCTCAACTCTCCAAATAGA CTCGAGTCTATTTGGAGAGTTGAGC -3'
